# Single‐cell RNA sequencing reveals cellular and molecular reprograming landscape of gliomas and lung cancer brain metastases

**DOI:** 10.1002/ctm2.1101

**Published:** 2022-11-06

**Authors:** He‐Fen Sun, Liang‐Dong Li, I‐Weng Lao, Xuan Li, Bao‐Jin Xu, Yi‐Qun Cao, Wei Jin

**Affiliations:** ^1^ Department of Breast Surgery Key Laboratory of Breast Cancer in Shanghai Fudan University Shanghai Cancer Center Shanghai China; ^2^ Department of Neurosurgery Fudan University Shanghai Cancer Center Shanghai China; ^3^ Department of Pathology Fudan University Shanghai Cancer Center Shanghai China; ^4^ Department of Oncology Shanghai Medical College Fudan University Shanghai China

**Keywords:** brain metastasis, glioma, lung adenocarcinoma, programmed cell death 1 ligand 1 (PDL1), single‐cell RNA sequencing, tumour microenvironment

## Abstract

**Background:**

Brain malignancies encompass gliomas and brain metastases originating from extracranial tumours including lung cancer. Approximately 50% of patients with lung adenocarcinoma (LUAD) will eventually develop brain metastases. However, the specific characteristics of gliomas and lung‐to‐brain metastases (LC) are largely unknown.

**Methods:**

We applied single‐cell RNA sequencing to profile immune and nonimmune cells in 4 glioma and 10 LC samples.

**Results:**

Our analysis revealed that tumour microenvironment (TME) cells are present in heterogeneous subpopulations. LC reprogramed cells into immune suppressed state, including microglia, macrophages, endothelial cells, and CD8^+^ T cells, with unique cell proportions and gene signatures. Particularly, we identified that a subset of macrophages was associated with poor prognosis. ROS (reactive oxygen species)‐producing neutrophils was found to participant in angiogenesis. Furthermore, endothelial cells participated in active communication with fibroblasts. Metastatic epithelial cells exhibited high heterogeneity in chromosomal instability (CIN) and cell population.

**Conclusions:**

Our findings provide a comprehensive understanding of the heterogenicity of the tumor microenvironment and tumour cells and it will be crucial for successful immunotherapy development for brain metastasis of lung cancer.

## Graphical abstract

1. Single‐cell transcriptional profiles in gliomas and lung‐to‐brain metastases.
2. TAMs (macrophage and microglia) exhibited phenotypic and functional diversity.
3. Endothelial cells communicated with neutrophils or fibroblasts to support angiogenesis.
4. Metastatic epithelial cells exhibited higher chromosomal instability and enriched a subpopulation with stem cell‐like phenotype.

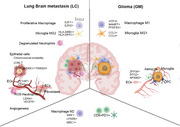

## 1 INTRODUCTION

Brain malignancies include the primary tumours, such as glioma, and brain metastases which originated from extracranial primary tumours, including lung cancers.^1^ Lung adenocarcinoma (LUAD) is the most common type of non‐small‐cell lung cancer (NSCLC). Approximately 50% of patients with LUAD will eventually develop brain metastases. Patients diagnosed with brain metastases usually have a short survival time.^1^ Given the current limited treatment strategies for these patients, it is pivotal to have a deep comprehensive understanding of metastatic lung cancer and its associated microenvironments. Particularly, an in‐depth understanding of the microenvironment heterogeneity between lung cancer brain metastases and primary brain tumours will aid the development of precise treatments for different brain tumours.

Immune checkpoint blockade (ICB) agents, such as programmed cell death 1 (PD1) or PDL1 inhibitors, have been recommended as the first‐line treatment in many patients with advanced LUAD.^2^, ^3^ However, extensive challenges exist, such as primary and acquired resistance, the lack of predictive and prognostic biomarkers, and treatment‐related adverse effects,^4^ the particularity and “immune privilege” feature of brain TME.^5^ Further understanding of the immune‐suppressive components of the brain tumour microenvironment (TME) can be exploited for novel immunotherapy strategies.

Epidermal growth factor receptor (EGFR) mutation results in aberrant activation of kinase signalling and occurs in approximately 15% of NSCLC cases.^6^ Patients diagnosed with EGFR mutations have a good initial clinical response to EGFR tyrosine kinase inhibitors; however, the disease will eventually progress due to acquired drug resistance.^7^, ^8^, ^9^ Further understanding the diversity of tumour cells with different EGFR statuses may promote the improvement of clinical treatment of lung metastasis patients with tyrosine kinase inhibitors.

To provide more detailed insight into the traits of the TME of LUAD metastases and glioma (GM), we performed single‐cell RNA sequencing (scRNA‐seq) on fresh brain resection samples of GM and lung‐to‐brain metastases (LC). Our study revealed the difference in immune cell composition and evaluated the function and genetic heterogeneity of the TME in GM and LC groups. We also analysed the heterogeneity of tumour cells in the LC group and found a group of stem cell‐like cells with specific molecular markers, suggesting possible drug targets.

## 2 MATERIALS AND METHODS

### 2.1 Human specimens and ethical approval

The brain tumour samples were collected from patients at Shanghai Cancer Center. Sample detailed information were listed in Tables S1 and S2. This study was approved by the Ethics Committee of Fudan University Shanghai Cancer Center (FUSCC), and each participant signed an informed consent document.

### 2.2 Single‐cell dissociation

The brain tumour tissues were resected and kept in MACS tissue storage solution (Miltenyi Biotec). Tissue samples were washed with PBS (phosphate‐buffered saline), cut into small pieces on ice and digested with tumour dissociation kit (Miltenyi Biotec). Then, samples were passed through a 70 μm cell strainer and centrifuged for 5 min at 300 g. Red blood cell lysis buffer (Miltenyi Biotec) was used to lyse red blood cells in the pelleted cells. Cell pellets were washed with and re‐suspended in 0.04% BSA in PBS and passed through a 35 μm cell strainer. Single cells were then stained with Calcein‐AM (Thermo Fisher Scientific) and Draq7 (BD Biosciences) to evaluate cell viability.

### 2.3 Single‐cell RNA sequencing

The transcriptomic information of the single cells (14 samples) was obtained by performing the BD Rhapsody system. Single‐cell capture was achieved by a random distribution of a single‐cell suspension across >2 00 000 microwells. Beads with oligonucleotide barcodes were saturated to make sure per bead binds with per cell. The cells were lysed and the mRNA molecules were hybridised to barcoded capture oligos on the beads. Beads were then collected with separate tube to reverse transcription and ExoI digestion. After cDNA synthesis, each cDNA molecule was labelled with a cell barcode and a unique molecular identifier (UMI) on the 5′ end. The transcriptome libraries were prepared using the BD Rhapsody single‐cell whole‐transcriptome amplification workflow. The libraries were quantified and sequenced (Illumina, San Diego, CA).

### 2.4 Single‐cell RNA statistical analysis

scRNA‐seq data analysis was performed by NovelBio Bio‐Pharm Technology Co., Ltd. The clean data was obtained by applying fastp with the default parameter to filter the adaptor sequence and deleted the low‐quality reads. The UMI‐based clean data were mapped to the human genome (Ensembl version 91)^10^ utilising STAR mapping with customised parameters. Seurat package (version: 3.1.4, https://satijalab.org/seurat/)^11^ was used for cell normalisation and regression. Mutual nearest neighbour (MNN) was applied to eliminate the potential batch effect. Subsequently, the top 10 principals were used for Uniform Manifold Approximation and Projection (UMAP) or t‐distributed Stochastic Neighbor Embedding (tSNE) construction.

Utilising graph‐based cluster method (resolution = 0.8), we got the unsupervised cell cluster results based on the top 10 principals and the marker genes by FindAllMarkers function with Wilcoxon rank sum test algorithm with lnFC > 0.25, min.pct > 0.1and *p* value <.05.

### 2.5 Pseudo‐time analysis

Single‐Cell Trajectories analysis was performed withMonocle2 (http://cole‐trapnell‐lab.github.io/monocle‐release) using default parameter and DDR‐Tree. Branch fate‐determined gene analysis was performed by branch expression analysis modelling.

### 2.6 CytoTRACE analysis

Cellular Trajectory Reconstruction Analysis using gene Counts and Expression (CytoTRACE) is used to predict the relative differentiation state of cells (https://cytotrace.stanford.edu/).

### 2.7 Cell communication analysis

Cell communication analysis was based on the CellPhoneDB which is a public repository of the interactions of receptors and ligands. The normalised cell matrix was achieved by Seurat Normalization. Cell Communication significance (*p* value < .05) and significant mean was calculated based on the interaction.

### 2.8 SCENIC analysis

Single‐cell regulatory network inference and clustering (pySCENIC, v0.9.5)^12^ workflow was applied to investigate the regulation strength of transcription factors. The 20‐thousand motifs database was used for RcisTarget and GRNboost.

### 2.9 QuSAGE analysis (gene enrichment analysis)

The R package Quantitative Set Analysis for Gene Expression (QuSAGE) (2.16.1) analysis^13^ was used to analyse the relative activation of a given gene set such as pathway activation, the Kyoto Encyclopedia of Genes and Genomes (KEGG) pathways and metabolism pathways.^14^

### 2.10 Differential gene expression analysis

Function FindMarkers with Wilcoxon rank sum test algorithm was used to determine differentially expressed genes among samples. The criteria were: lnFC > 0.25, *p* value < .05 and min.pct > 0.1.

### 2.11 Co‐regulation gene analysis

Find_gene_modules function of monocle3^15^ was performed to discover the gene co‐regulation network.

### 2.12 Copy number variation estimation

Endothelial cells were used as a reference to analyse somatic copy number variations (CNVs) with the R package infercnv (v0.8.2). The extent of the CNV signal of each cell was scored, defined as the mean of squares of CNV values across the genome. Cells with a CNV signal above 0.05 and a CNV correlation above 0.5 were defined as putative malignant cells.

### 2.13 Survival analysis

We applied the TCGAbiolinks package^16^ based on the marker gene or marker gene set achieved from scRNA‐Seq together with the LUAD, low‐grade glioma (LGG) and glioblastoma multiform (GBM) expression profile and clinical information in the TCGA database for survival analysis. Furthermore, for gene set analysis, z‐score normalisation was applied based on the TCGA matrix and the average z‐score was calculated according to the gene set list for survival analysis.

### 2.14 Multiplexed immunohistochemistry

Whole tissue sections from LC03 and LC07 (4‐μm‐thick formalin‐fixed, paraffin‐embedded) were stained with primary antibodies (EpCAM (#2929, CST, 1:500), CD163(#93498, CST, 1:200), Topoisomerase IIα (#12286, CST,1:400), TRIM29(17542‐1‐AP, Proteintech, 1:100), DKK1(Abcam, ab109416, 1:800) sequentially and paired with TSA 7‐colour kit (abs50015‐100T, Absinbio). The order of antibodies/fluorescent dyes was showed as following: anti‐ EpCAM/TSA 480, anti‐ CD163/TSA 780, anti‐TOPIIα/TSA 620, anti‐TRIM29/TSA 520, anti‐DKK1/TSA 570 and then by staining with DAPI (D1306; Thermo Fisher). Pictures were scanned with Aperio Versa 8 tissue imaging system (Leica). Images were analysed using Indica Halo software.

## 3 RESULTS

### 3.1 Identification of cell populations in gliomas and lung‐to‐brain metastases

**FIGURE 1:**
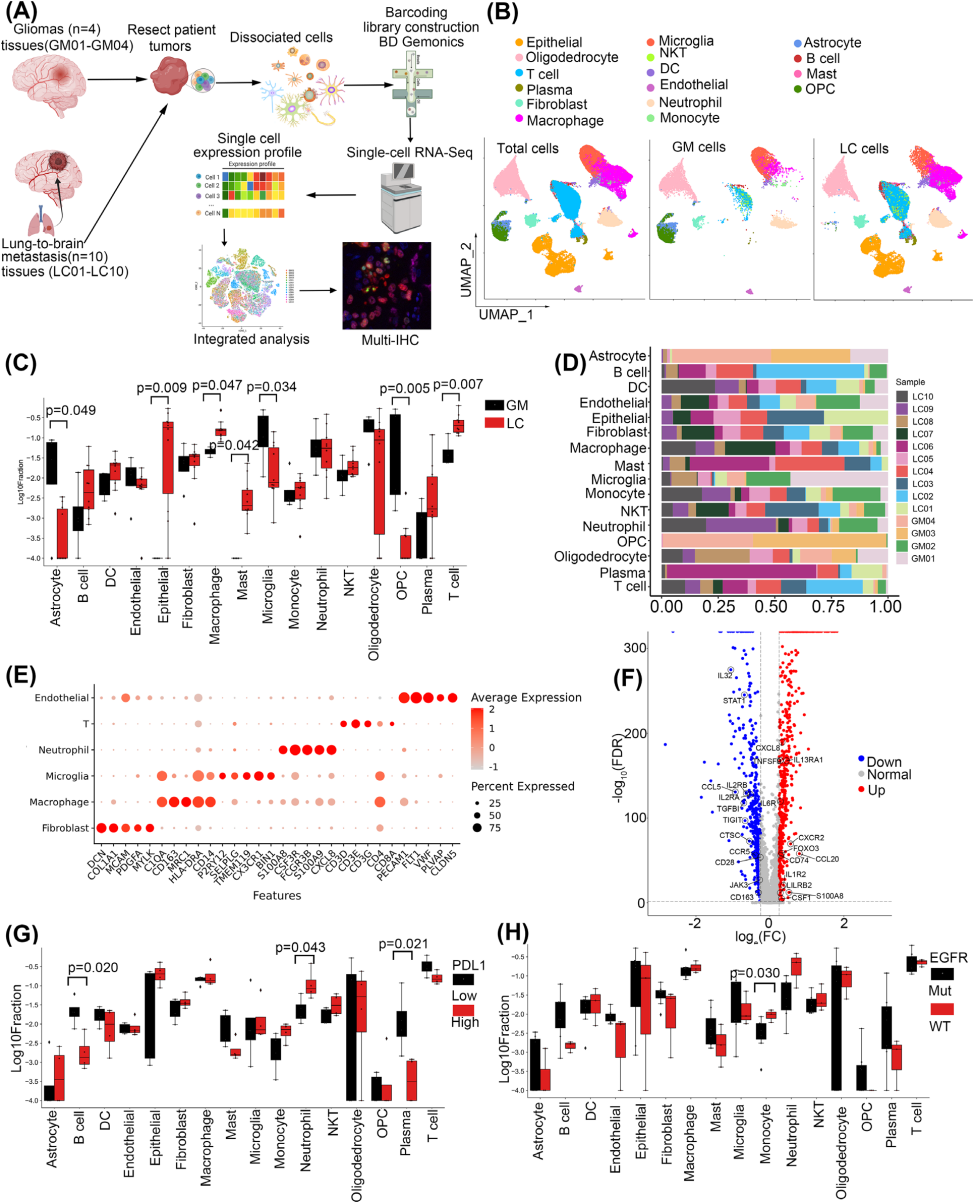
Overview of the single‐cell landscape for brain cancer. (A) Workflow showing sampling, sequencing and analysis process of clinical samples. (B) UMAP view of total cells obtained from 4 GM and 10 LC samples, colour‐coded by assigned cell type. (C) Cell type proportions in GM and LC, the black and red represented GM and LC, respectively. (D) Cell composition distribution for each patient sample. (E) Marker gene expression for each cell type, where dot size and colour represent the percentage of the marker gene. (F) The volcano plot of variable expression genes of TME between GM and LC. Upregulated genes (FC > 2) were coloured in red while downregulated genes (FC less than ‐ 2) were coloured in blue. (G) Cell type proportions in the PDL1 high (PDL1 expression > 1%) and low group, the black and red represented PDL1 low and PDL1 high, respectively. (H) Cell type proportions in EGFR mutation (mutation in Exon19, 20 or 21) and wild type group, the black and red represented EGFR mutation and wild type, respectively.

To explore the microenvironmental landscape and immune status in GM and LC, we profiled the transcriptomes of 61 867 individual cells obtained from 4 GM and 10 LC samples using scRNA‐seq (Figure 1A). The information on the data quality of scRNA‐seq was shown in Table S3. LC samples were further characterised into two groups according to EGFR mutation status or PDL1 expression. The detailed clinical information is summarised in Tables S1 and S2. We identified 28 clusters, including epithelial cells, endothelial cells, macrophages, microglial cells, astrocytes, immune cells (myeloid, T and B cells), and fibroblasts (Figure 1B).

The cell type proportions in GM and LC samples were analysed, and the results showed an abundance of invasion of immune cells, including T cells, mast cells and macrophages, in the LC group. Epithelial cells were distributed in the LC group, whereas astrocytes, microglia, and oligodendrocyte progenitor cells (OPCs) were found significantly more frequently in the GM group (Figure 1C; *p* < .05, Kruskal test). The cell type proportions in all the samples are shown (Figure 1D). Considering that malignant Oligodendrocyte/OPC and astrocyte were the composition of GM, we also compared the nonmalignant cell populations by CNV analysis between GM and LC samples in Figure S1A and the results were consistent with that in Figure 1C. The five specific marker genes of cell clusters that we were interested in based on their gene expression profiles are shown (Figure 1E). We also analysed the differentially expressed genes in the TME between GM and LC and found that 573 genes were downregulated and 522 genes were upregulated in the GM group (Figure 1F).

As PDL1 expression level and EGFR mutation status determined the treatment efficiency of anti‐immune therapy and TKIs (tyrosine kinase inhibitor) targeted therapy, we further evaluated the cell types distribution according to the expression of PDL1 or the status of EGFR. We grouped the LC samples into PDL1‐high (PDL1 expression >1%) and PDL1‐low (PDL1 expression ≤1%) or EGFR‐mutated and EGFR‐wild‐type (WT) samples. The cell proportions in different groups are shown. Among all the cell types, B cells, and plasma cells showed a significant increase while neutrophils were reduced in PDL1‐low samples (Figure 1G, *p* < .05, Kruskal test), and monocytes were notably increased in the EGFR‐WT group (Figure 1H; *p* < .05, Kruskal test).

### 3.2 A subset of proliferative macrophages is associated with poor prognosis

**FIGURE 2:**
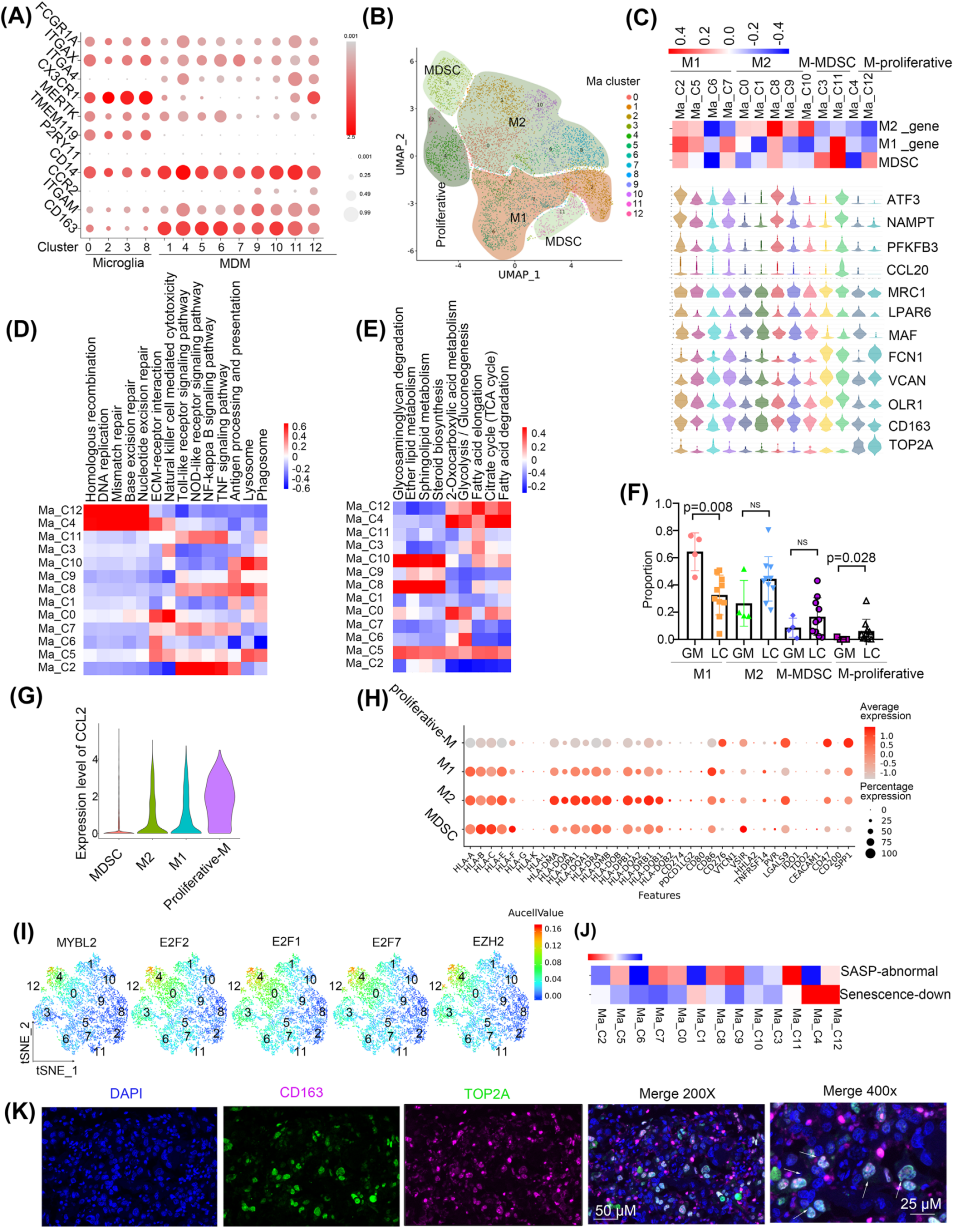
A subset of proliferative MDMs is associated with poor prognosis. (A) The bubble plot showed the gene expressions of the TAMs that originated from tissue‐invading monocyte‐derived macrophages (MDMs) or CNS‐resident microglia‐derived macrophages (microglia). (B) The UMAP view of macrophage. (C) Marker gene expression profiles of macrophage cell type. (D) KEGG pathway enriched in macrophage. Red colour represents upregulation and blue colour means downregulation. (E) Metabolism pathway enriched in macrophage. Red colour represents upregulation and blue colour means downregulation. (F) The relative proportion of M1 and M2 in GM and LC. (G) Expression of CCL2 in different types of macrophages showed with violin plot. (H) Gene bubble plot of immune checkpoint inhibition ligand genes. (I) Expression of genes was shown in the tSNE view. (J) Scoring all the macrophages by the senescence gene list. (K) Multiplex immunofluorescence staining of LC tissue.

Tumour‐associated macrophages (TAMs) originate from tissue‐invading monocyte‐derived macrophages (MDMs) or central nervous system (CNS)‐resident microglia‐derived macrophages. To determine the cell origin of the TAMs, we scored all the TAM clusters according to the following gene signatures: FCGR1A^+^/ITGAX^+^/ITGA4^−^/CX3CR1^+^/MERTK^+^ (CNS‐resident microglia), CD14^+^/CCR2^+^/ITGAM^+^ (monocytes), and ITGA4^+^/MERTK^+^/CD163^+^/FCGR1A^+^ (MDMs).^17^ The results showed that C0, C2, C3 and C8 cells might originate from CNS‐resident microglia, the others may originate from MDMs. To further confirm the existence of CNS‐resident microglia, we also checked the expression of canonical markers of microglia, such as TMEM119 and P2RY11. We found that TMEM119 was highly expressed in the CNS‐resident microglia, while P2RY11 was scarcely expressed in all the TAMs (Figure 2A). Then, we found that MDMs were present in high proportions in LC tissues, while microglia were abundant in GM tissues (Figure S1B). Our results indicated that the sources of TAM in metastases and primary tumours are different.

To explore the function of MDMs, we re‐clustered and stratified them into M1 macrophages (Ma_C2, C5, C6, and C7; ATF3^+^/NAMPT^+^/PFKFB3^+^/CCL20^+^), M2 macrophages (Ma_C0, C1, C8, C9, and C10; CD163^+^/MAF^+/^LPAR6^+^/MRC1^+^), and myeloid‐derived suppressor cells (MDSCs) (Ma_C3 and C11; OLR1^+^/VCAN^+^/FCN1^+^) (Table S4) based on unique marker expression after separating proliferative macrophages (Ma_C4 and C12; TOP2A^+^) (Figure 2B,C). Then, we found that M1 macrophages were enriched in immune‐activating pathways. Proliferative cells showed highly activated DNA replication and DNA repair pathways (Figure 2D). M2 macrophages showed activation of lipid metabolism processes, such as sphingolipid metabolism and steroid biosynthesis, while proliferative macrophages showed enrichment of energy metabolism processes, such as the citrate cycle (Figure 2E).

We determined the abundance of MDMs in GM and LC groups and found that the percentage of M1 was higher in GM (*p* = .008, Wilcoxon test), proliferative macrophages was lower in GM (*p* = .028, Wilcoxon test), and M2 macrophages were relatively higher in LC, albeit the difference was not statistically significant (*p* = .076, Wilcoxon test) (Figure 2F). In addition, M1 macrophages showed more enrichment of immune‐activating pathways such as the tumor necrosis factor (TNF) signalling pathway, and NF‐κB signalling pathway in GM than in LC, while M2 macrophages showed downregulation of pathways related to phagosomes, regulation of the actin cytoskeleton in GM compared with LC. (Figure S1C,D)

We next analysed cytokine expression in MDMs and found that proliferative clusters highly expressed CCL2 (Figure 2G), which has been reported to promote M2 macrophage polarisation.^18^ M2 and proliferative macrophages had increased expression of the immune checkpoint inhibitory ligand genes CD276, LGALS9, and CD47 and reduced expression of CD86, which is a checkpoint activating ligand gene (Figure 2H).

Most importantly, proliferative cells showed enrichment of several transcription factors that function in cell proliferation, such as MYBL2, EZH2, E2F1, E2F2, and E2F7 (Figure 2I and Figure S1E). It has been reported that the E2F7‐EZH2 axis regulates glioblastoma progression.^19^ The absence of E2F1 and E2F2 initiates the senescence program in macrophages^20^; consistently, we found that proliferative macrophages had downregulated senescence‐associated processes (Figure 2J and Figure S1F–H). Finally, we verified the cell populations by performing multiplex immunofluorescence staining for the marker genes TOP2A and CD163 (Figure 2K).

Moreover, to investigate the prognostic value of these clusters in LUAD, LGG or GBM, we analysed their gene signatures in The Cancer Genome Atlas (TCGA). Interestingly, the results showed that the proliferative group was related to poor prognosis in both LUAD and LGG but had no prognostic significance in GBM (Figure S1I,J and Table S5).

### 3.3 Microglia exhibited multiple polarisation phenotypes in the brain

**FIGURE 3:**
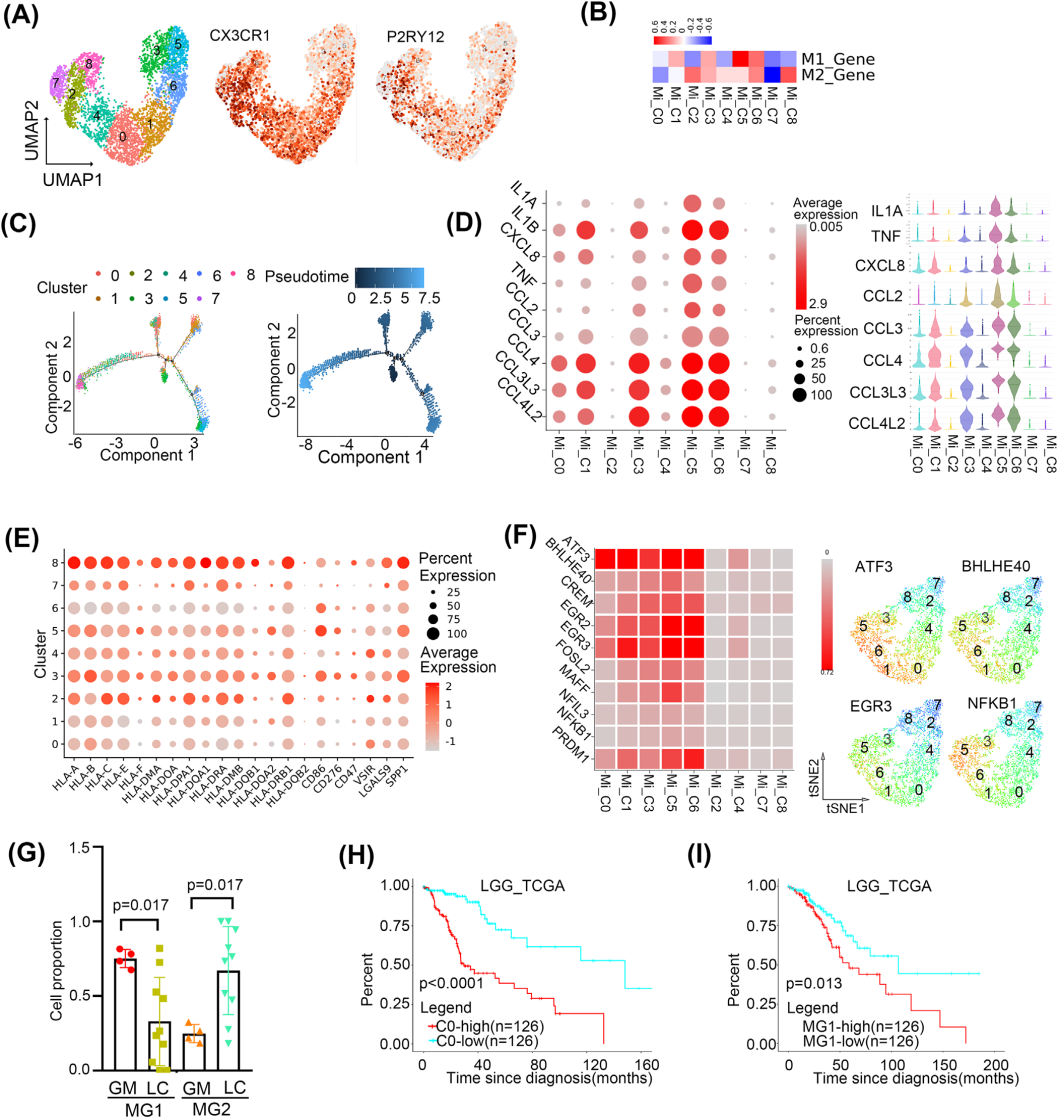
Microglia exhibited multiple polarisation phenotypes in the brain. (A) The UMAP view of microglia with the expression of CX3CR1 and P2RY12. (B) Scoring all the microglia by the macrophage phenotyping and polarisation gene list. (C) Differentiation trajectory of microglia, with each colour coded for clusters (left) and pseudo‐time (right). (D) Bubble plot of the cytokine expression in microglia, colour and dot size represented the average and percent expression, respectively (The left panel). The violin plots of the cytokine expression in microglia (The right panel) (E) Bubble plot of immune checkpoint inhibition ligand gene. (F) The expression of indicated transcriptional factors showed with heatmap (The left panel) and UMAP views (The right panel). (G) The relative proportion of MG1 and MG2 in GM and LC. (H) The survival curve of cluster 0 gene signature in LGG in TCGA. (I) The survival curve of MG1 gene signature in LGG in TCGA.

We then sub‐clustered CNS‐resident microglia‐derived macrophages into nine clusters according to gene expression profiles (Figure 3A and Figure S2A). All the clusters were found in both GM and LC (Figure S2B). In view of the similar polarisation of microglia and macrophages, we evaluated the polarisation phenotype of microglia according to lists (Table S4) of genes used for macrophage phenotyping (related to M1 and M2 polarisation). A distinct M1‐type signature was observed in Mi_C1 and Mi_C5, while an M2‐type signature was enriched in Mi_C2, Mi_C4 and Mi_C8 (Figure 3B). Of note, not all clusters could be simply classified according to the M1 and M2 gene signatures. To further understand the phenotype, we next inferred the differentiation trajectory of these cells by pseudo‐time analysis. The results showed that Mi_C3 may include cells in the initial state and Mi_C0, C1 may include cells in the intermediate state while Mi_C5/C6 and Mi_C2/C4/C6/C8 were towards two different directions in the terminal state (Figure 3C). Cytokine secretion and MHCII expression were the main features of activated microglia.^21^ We found that Mi_C0, C1, C3, C5 and C6 had increased expression of pro‐inflammatory cytokines such as IL1A, IL1B, TNF, CCL3, CCL4, CCL3L3, CCL4L2 and CXCL8. However, Mi_C2, C4, C7 and C8 scarcely expressed these cytokines (Figure 3D). Mi_C2, C4, C7 and C8 highly expressed MHCII genes such as HLA‐DRB1 and HLA‐DPA1 (Figure 3E). Therefore, we clarified microglia into cytokine‐secreted MG1 (CX3CR1^+^/CCL3^+^/CCL4^+^, Mi_C0, C1, C3, C5 and C6) and MHCII highly expressed MG2 (CX3CR1^+^/HLA‐DRB1^+^/HLA‐DPA1^+^, Mi_C2, C4, C7 and C8).

Next, we found that MG1 microglia highly expressed immune checkpoint activated ligand gene CD86 and MG2 highly expressed immune checkpoint inhibited ligand genes LGALS9 and VISR (Figure 3E). SCENIC analysed results also confirmed MG1 microglia highly expressed pro‐immune related translational factors such as ATF3,^22^ BHLHE40,^23^ EGR2/EGR3^24^ and NFKB1^25^ (Figure 3F). These results confirmed that microglia exhibited two polarisation phenotypes.

We then found that MG1 microglia were obviously abundant in GM, while MG2 microglia accounted for a much higher percentage in LC (*p* < .05, Wilcoxon test) (Figure 3G). Besides, GO analysis showed that MG1 in GM was enrichment with protein folding while MG2 in LC was antigen processing and presentation and immune response (Figure S2D,E). In particular, Mi_C1 and Mi_C2 presented higher proportions in LC and GM, respectively (*p* < .05, respectively, Wilcoxon test) (Figure S2C). Mi_C1 and C2 had enrichment of the opposite metabolic and KEGG pathways (Figure S2F).

We investigated the association of gene signatures of microglia with the prognosis of LGG and GBM in TCGA. Only Mi_C0 and MG1 gene signatures were associated with a poor prognosis in LGG (*p* < .0001, *p* = .013, respectively, Cox regression) (Figure 3H,I) and no clusters indicated a significant association of prognosis in GBM (Table S6).

### 3.4 ROS‐producing neutrophils may participate in tumour angiogenesis in brain malignancies

**FIGURE 4:**
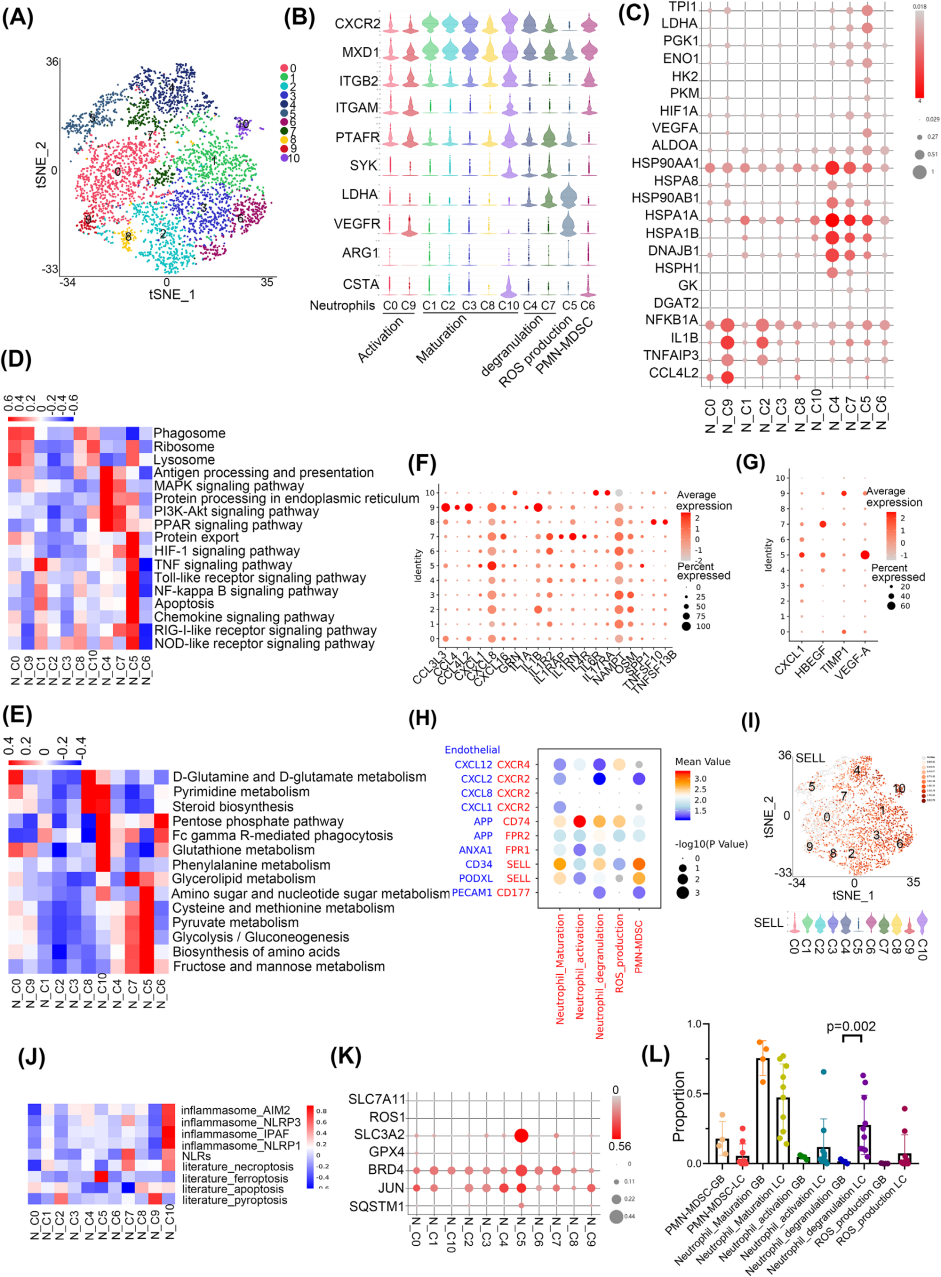
ROS‐productive neutrophils may participate in tumour angiogenesis in brain malignancies. (A) The tSNE view of neutrophils. (B) Violin plots of the expression of marker gene of neutrophil subtypes. (C) Bubble plot of gene expression in neutrophils. (D) KEGG pathway enriched in neutrophils. Red colour represents upregulation and blue colour means downregulation. (E) Metabolism pathway enriched in neutrophils. Red colour represents upregulation and blue colour means downregulation. (F) The bubble plot showed the expression of cytokines in neutrophils. (G) The bubble plot showed the expression of growth factors in neutrophils. (H) The bubble plot showed the interaction between neutrophils with ECs. Blue represents the ligand in ECs, red represents the receptor in neutrophils. (I) Expression of SELL in neutrophils was shown in the tSNE view (the up panel) and violin plot (the down panel). (J) The inflammatory cell death pathways of neutrophils. (K) The bubble plot showed the expression of ferroptosis‐related genes in neutrophils. (L) The relative proportion of the different types of neutrophils in GM and LC.

The 11clusters of neutrophils were identified as PMN‐MDSCs (polymorphonuclear myeloid‐derived suppressor cells) (N_C6; AGE1^+^CSTA^+^), mature neutrophils (N_C1, C2, C3, C8 and C10; CXCR2^+^MXD1^+^), activated neutrophils (N_C0 and C9; ITGB2^+^ITGAM^+^), ROS‐producing neutrophils (N_C5; LDHA^+^VEGFR^+^) and degranulated neutrophils (N_C4 and C7) by QuSAGE analysis (Figure 4A,B). Activated neutrophils were enriched with NF‐κB and Toll‐like receptor signalling pathways (NFKBIA and TNFAIP3). Degranulated neutrophils showed activation of antigen processing and presentation (HSP90AA1, HSPA8 and HSPA1A). In addition, module 34 showed that N_C4 was enriched in the FOXO signalling pathway, which regulates neutrophil survival and degranulation as an unfolded protein response, confirming that N_C4 cells were degranulated (Figure S3A). ROS‐producing neutrophils showed upregulation of the HIF‐1 signalling pathway with an expression of HIF1A, LDHA, and VEGF‐A. LDHA has been reported to mediate ROS production.^26^ HIF1A functions in angiogenesis, cell survival, glucose metabolism and invasion.^27^ Metabolic analysis also showed that ROS‐producing neutrophil‐activated glycolysis and fructose and mannose metabolism (TPI1, LDHA, PGK1, ENO1, HK2, and PKM) (Figure 4C–E).

Chemokines are known to regulate the migration, tumour infiltration and function of neutrophils.^28^ Therefore, we further analysed the expression of chemokines in neutrophils and found that activated neutrophils showed high expression of CCL3L3, CCL4L2 and degranulated neutrophils showed increased expression of IL‐4R, which may suppress neutrophil recruitment, chemotaxis, and effector functions^29^ and regulate the expression of SYK^30^ (Figure 4F). ROS production neutrophils showed increased expression of CXCL8 and VEGFA, which was consistent with our results that they may play an important role in angiogenesis (Figure 4F,G).

Our results indicated that neutrophils may participate in angiogenesis, hence we wonder whether neutrophils can communicate with ECs. Cell communication analysis showed that CD74 (in activated, degranulated and ROS productive neutrophils) and the ligand APP in ECs, while SELL (in mature, degranulated neutrophils and PMN‐MDSCs) and the ligands CD34/PODXL in ECs may form unique interaction pairs (Figure 4H). ROS productive neutrophils secreted VEGFA, which was consistent with our results that they may play an important role in angiogenesis (Figure 4G). In addition, SELL, which was reported to regulate human neutrophil transendothelial migration,^31^ was highly expressed in mature, degranulated neutrophils and PMN‐MDSCs (Figure 4I). We also performed the CSOmap algorithm^32^ to investigate the three‐dimensional pseudo‐space based on cell expression profiles of neutrophils and ECs cluster (Figure S3B). The results confirmed neutrophils and ECs formed tight primary linked structures (Figure S3C,D) and were close to each other in pseudo‐space (Figure S3E).

Since the regulation of neutrophil death ways played a crucial role in homeostasis and inflammatory states,^33^ we further explored the inflammatory cell death pathways of neutrophils. The results showed that ROS productive neutrophils had enrichment of ferroptosis and elevated expression of genes that suppressed ferroptosis, such as SLC3A2,^34^ BRD4,^35^ JUN, and SQSTM1.^36^ Neutrophil‐induced ferroptosis has been reported to promote tumour necrosis^37^ (Figure 4J,K). Degranulated neutrophil C7 and mature neutrophil C10 showed the expression of necroptosis related gene TLR4, CFLAR, and MLKL, while activated neutrophil C9 showed the expression of pyroptosis‐related gene IL1B (Figure S3F,G).

To evaluate the different cell types in LC and GM, we determined the percentage of neutrophil types and found that degranulated neutrophils had a higher proportion in LC than in GM (*p* = .002, Wilcoxon) (Figure 4L). Next, we found that degranulated neutrophils had upregulation of the VEGF signalling pathway and chemokine signalling pathway but downregulation of HIF‐1 signalling and glycolysis pathway in GM than LC (Figure S3H). We further investigated the characteristics of N_C4 and N_C7, and the results showed that the expression of genes such as FOXO3, NOTCH and DTX4, which are involved in the NOTCH signalling pathway, were highly increased in LC (Figure S3I).

### 3.5 Endothelial cells have tissue specificity and communicate with tumour‐associated fibroblasts

**FIGURE 5:**
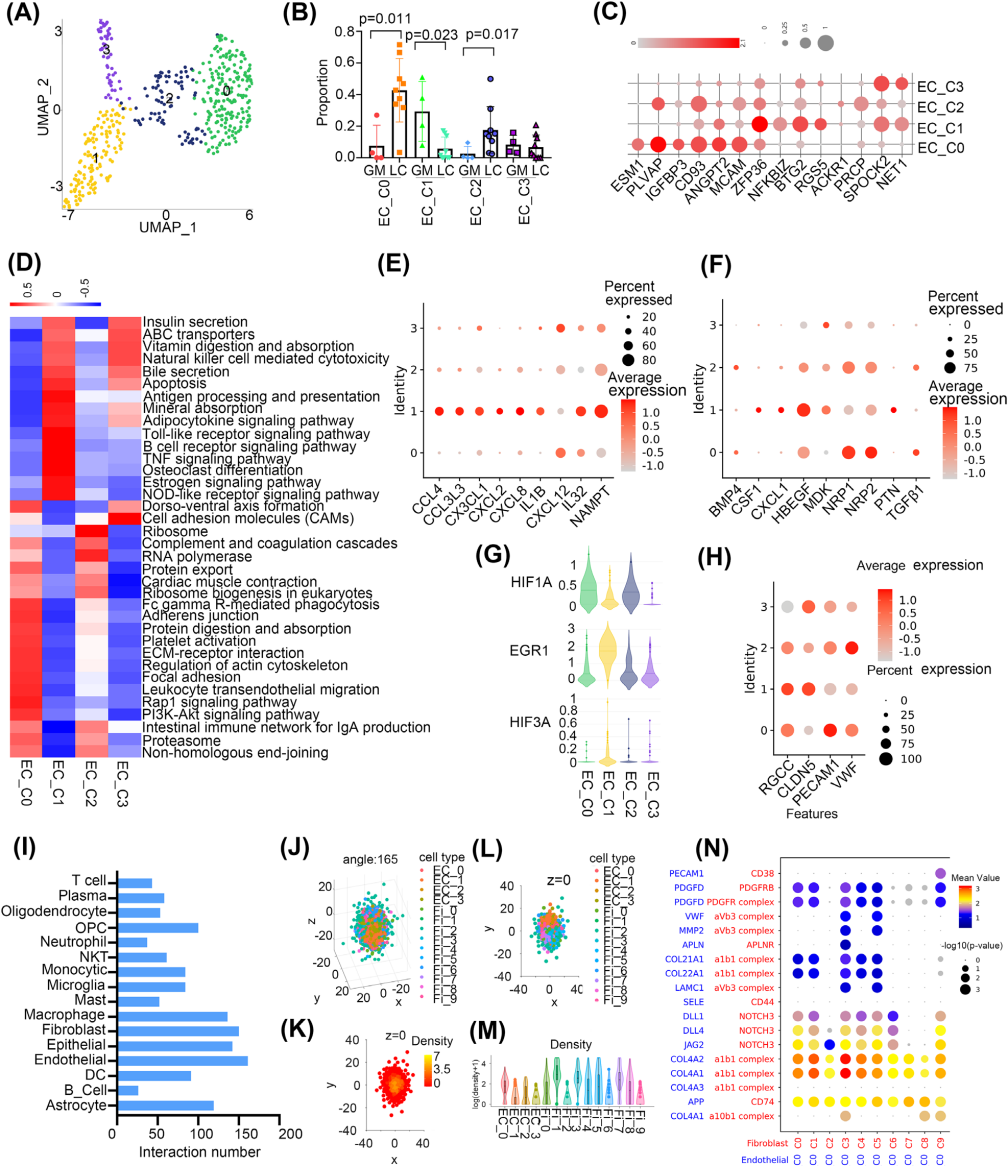
Endothelial cells communicate with tumour‐associated fibroblast cells. (A) The UMAP view of ECs. (B) The relative proportion of ECs in GM and LC. (C) Marker gene expression profiles of ECs showed with bubble plots. (D) KEGG pathway enriched in ECs. Red colour represents upregulation and blue colour means downregulation. (E) The bubble plot showed the expression of cytokines in ECs. (F) The bubble plot showed the expression of growth factors in ECs. (G) The expression of EGR1, HIF3A and HIF1A in ECs. (H) Bubble plots showed the expression of endothelial function‐associated genes. (I) Numbers of inferred interactions between ECs with other cell types. (J) Spatial organisation (angle = 165) of fibroblast and ECs in the pseudo‐space inferred by CSOmap based on the scRNA‐seq data. Each dot represents a cell, and its colour represents the corresponding cell state. (K) The cross‐section of z = 0 of the pseudo‐space. The colour of the dots represents cell density. (L) Location of fibroblast and ECs in the cross‐section of pseudo‐space z = 0. (M) The difference in cell density between cell clusters of fibroblast and ECs. (N) The bubble plot showed the interaction between C0 in ECs with fibroblast clusters. Blue represents the ligand in ECs, red represents the receptor in fibroblast.

We identified four cell clusters of endothelial cells (ECs) after removing low‐quality cells (Figure 5A). The expression of KDR (VEGFR1) and lack of PDPN expression indicated that the ECs were derived from vascular rather than lymphatic vessels (Figure S4A). Then we found that EC_C1 was abundant in GM, while EC_C0 and C2 were increased in LC (*p* < .05, Wilcoxon test) (Figure 5B). ECs showed activation of cell junction‐related pathways such as extracellular matrix (ECM)–receptor interactions, regulation of the actin cytoskeleton, focal adhesion and leukocyte transendothelial migration in LC compared with GM, while ECs in GM‐enriched immune‐related pathways such as protein processing in the endoplasmic reticulum, antigen processing and presentation and MAPK signalling pathway (Figure S4B). Our results indicated that different subgroups of ECs may have tissue specificity.

Then, we found that LC‐enriched EC_C0 and C2 showed increased expression of genes that promoted angiogenesis (PLVAP, and CD93) and activated cell junction‐related pathways and downregulated immune‐related pathways, such as the B cell receptor signalling pathway and antigen processing and presentation. They also showed high expression of MCAM, which might be involved in the recruitment of activated T cells to inflammation sites.^38^ GM‐enriched EC_C1 expressed immune response associated genes (ZFP36, NFKBIZ, BTG2) and showed enrichment of immune‐related pathways, such as the TNF signalling pathway, which functioned in endothelial barrier disruption^39^ (Figure 5C,D). Module 45 showed that EC_C1 was enriched FOXO signalling which plays essential roles in suppressing EC growth and tube morphogenesis (Figure S4C)^40^ and limiting vascular expansion.^41^ The metabolism analysis showed that EC_C0 and C2 were enriched in energy metabolic pathways such as glycolysis/gluconeogenesis, the pentose phosphate pathway and oxidative phosphorylation, while EC_C1 showed high activation of steroid biosynthesis and thiamine metabolism (Figure S4D).

To further investigate whether ECs had a different distribution of chemokines and growth factors which can regulate the function of ECs, we analysed their expression profiles in different cell clusters. As expected, EC_C0/C2 and C1 had different cytokine expression levels. For instance, EC_C0/C2 showed elevated expression of CXCL12, NRP1, and NRP2, whereas EC_C1 showed increased expression of CXCL8, CXCL4, NAMPT and HBEGF (Figure 5E,F). Hypoxia remodels the endothelial phenotype and alters the expression of EGR1 and HIF, which coordinate separate activation of distinct genes.^42^ In our study, we found that EC_C1 had increased expression of EGR1 and HIF3A, while EC_C0/C2 had elevated expression of HIF1A (Figure 5G). Furthermore, EC_C0/C2 expressed VWF and PECAM‐1, which is an essential protein in transendothelial migration.^43^ EC_C1 had increased expression of CLDN5, which participates in the regulation of the endothelial barrier^44^ (Figure 5H).

Moreover, we investigated cell‐cell interactions with CellPhoneDB.^45^ In particular, ECs had higher numbers of inferred interactions with fibroblasts, ECs and macrophages (Figure 5I). In addition, EC_C0, C1 and C2 represented a large portion of cells interacting with fibroblasts (Figure S4E). We also performed the CSOmap algorithm to investigate the three‐dimensional pseudo‐space based on cell expression profiles of fibroblasts and ECs cluster and the results confirmed fibroblasts and EC cells formed tight primary linked structures and were closed to each other in pseudo‐space (Figure 5J–M). Fibroblasts were divided into cancer‐associated fibroblasts (CAFs); (Fi_C7 and C8) and myofibroblasts (Fi_C1‐C6 and Fi_C9) (Figure S4F). We found that EC_C0 had stronger gene interactions with all fibroblasts and the interaction between EC_C0 and fibroblasts showed stronger pairs of COL4A2 and COL4A1 on ECs and the receptor a1b1 complex on myofibroblasts and JAG2 and DLL4 on ECs and their receptor NOTCH3 on myofibroblasts (Figure 5N). Myofibroblasts have been reported to promote tissue remodelling, tumour stroma remodelling^46^ and angiogenesis.^47^ We also performed H&E staining to evaluate the spatial proximity of fibroblasts and ECs and the results showed that fibroblasts were tightly around the EC cell in the blood vessel inside the tumour but we did not see this in the blood vessel around the tumour (Figure S4G). In conclusion, cellular interactions between ECs and fibroblasts help to promote angiogenesis in LUAD and brain metastases.^48^

### 3.6 Heterogeneity of T cells and the association with PDL1 expression

**FIGURE 6:**
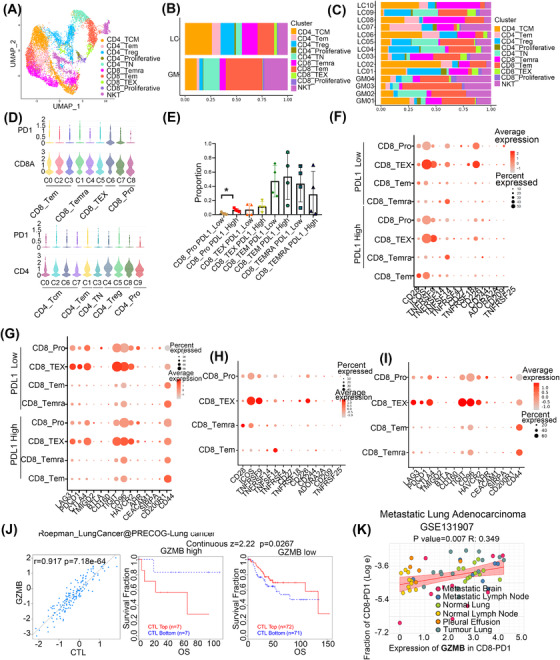
The heterogeneity for T cells and the association with the expression of PDL1. (A) The UMAP view of different types of T cells. (B) The relative proportion of the different types of T cells in LC and GM. (C) The relative proportion of the different types of T cells in each patient of LC and GM. (D) Violin plots showed the expression of PD1 in CD4^+^ and CD8^+^ subtypes. (E) The relative proportion of subtype of CD8^+^ T cells in PDL1 high and low expression group. (F) Bubble plots showed the expression of the immune checkpoint activation receptor gene of the subtype of CD8^+^ T cells in the PDL1 high and low expression group. (G) Bubble plots showed the expression of the immune checkpoint inhibition receptor gene of the subtype of CD8^+^ T cells in the PDL1 high and low expression group. (H) Bubble plots showed the expression of immune checkpoint activation receptor genes of a subtype of CD8^+^ T cells. (I) Bubble plots showed the expression of immune checkpoint inhibition receptor genes of a subtype of CD8^+^ T cells. (J) Correlation between the gene expression of GZMB and the infiltration of CTLs was shown on the left and the Kaplan–Meier plots of overall survival (OS) for LUAD patients with CTLs with the top and bottom TIDE prediction scores based on gene expression of GZMB (on the right). The *p* value was calculated by testing the association between TIDE prediction scores and overall survival with the two‐sided Wald test in a Cox‐PH regression. (K) Correlation of expression of GZMB in CD8^+^ PD1^+^ T cells with the fraction of CD8^+^ PD1^+^ T cells in metastatic LUAD.

T cells identified in this study were classified into CD8^+^ T (effector memory T (TEM), central memory T (TCM), and terminally differentiated effector T (Temra)), CD4^+^ T (naive, TCM, and regulatory), natural killer T (NKT), and proliferative T cells (Figure 6A). Then, we found less abundant CD4 TCM and CD8^+^ Temra cells and more NKT and CD8+ TEM cells in LC tumour tissue compared to GM samples. CD8^+^ exhausted T (TEX) and CD4^+^ TEM cells were mainly seen in LC, though the difference had no significance (Figure 6B,C). In general, the difference in cellular proportions and gene signatures indicates that metastatic tumour tissue has reprogrammed T cells.

PD1 was expressed in several immune cells such as T cells. The interaction of PD1 with its ligand PDL1 is involved in suppressing anti‐tumour immunity response. Therefore, we wondered whether some subtypes of CD4^+^ or CD8^+^ T cells may alter the anti‐tumour efficacy with anti‐PD1 or PDL1 treatment. We first subtyped CD8^+^ and CD4^+^ T cells based on the expression of marker genes (Figure S5A,B). Then, the expression of PD1 in these cells was analysed and the results showed that CD8^+^ TEX cells and proliferative CD8^+^ T cells were PD1 high expression (Figure 6D). We further investigated whether CD8^+^ T cell proportions or functions were altered with PDL1 expression in tumour cells and found that the abundance of proliferative CD8^+^ cells was increased in the PDL1‐high expression group, but the percentages of other CD8^+^ T cell subtypes were not significantly different (Figure 6E). Interestingly, in proliferative CD8^+^ cells, we also observed higher expression of the immune checkpoint inhibition receptor genes LAG3, AHR3, and TIGIT and the immune checkpoint activation receptor gene CD226 and lower expression of CD44 in the PDL1‐low group than in the PDL1‐high group (Figure 6F,G). In addition, we evaluated the expression of immune checkpoints in other CD8^+^ T cells subtypes and found that CD8^+^ TEX cells had high expression of the inhibitory receptor genes PDCD1, LAG3, and TIGIT (Figure 6H,I). Our results indicated that tumour immune therapy with an anti‐PD1/PDL1 inhibitor combined with other ICB agents may increase the effectiveness of treatments for cancer.

To investigate whether CD8^+^ T cells identified in our study can predict tumour immune evasion and immune therapy resistance in NSCLC, we evaluated the expression of their main marker genes of CD8+ cytolytic T lymphocytes (CTLs) in the patients who received anti‐CTLA4 or anti‐PD1 therapy in scTIME Portal.^49^ The results showed that the expression of GZMB (CD8_TEX), GZMH and PRF1 (CD8_Temra) was positively related to the proportion of CTLs. In addition, the higher expression of these genes indicated the poor prognosis of higher infiltration of CTLs and indicated that these genes may involve in the dysfunction of CTLs (Figure 6J and Figure S5C–E). We also used some single‐cell sequencing data in the public database with metastatic LUAD (including brain metastasis) to verify the effect of expression of GZMB, GZMH and PRF1 in the infiltration of PD1^+^CD8^+^ T cells and the results showed that the high expression of these genes was positively associated with the infiltration of PD1^+^CD8^+^ T cells (Figure 6K and Figure S5F–J). These results indicated that CD8_TEX and CD8_Temra may express genes that impaired the function of CTLs and increase the infiltration of PD1^+^CD8^+^ T cells to suppress the anti‐cancer immune response.

### 3.7 One subpopulation of cancer stem‐like cells was enriched in epithelial cells

We detected 7321 epithelial cells by the expression of EPCAM, KRT18 and KRT19 (Figure S6A). Next, by performing copy number alteration (CNA) analysis, malignant cells were defined as those with CNV signals above 0.05 and CNV correlations above 0.5. We identified 5533 malignant cells and categorised them into 10 sub‐clusters (Figure S6B). The inferred CNAs were canonical LC genome alterations,^50^ including gains of chromosomes 1 and 7 and losses of chromosomes 10 and 11 (Figure S6C).

**FIGURE 7:**
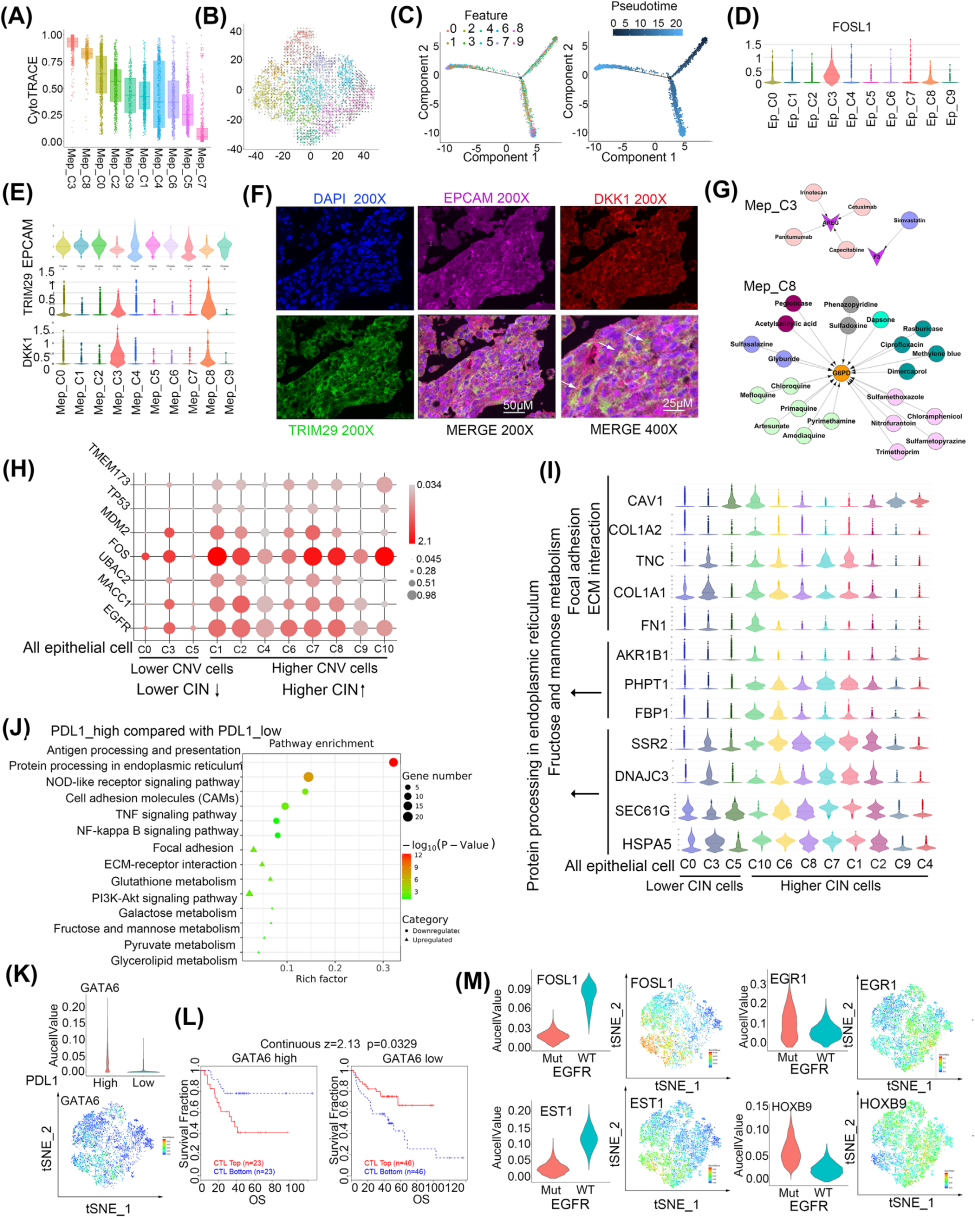
Epithelial cells harbour a subset of cancer stem cells and show heterogeneity of DNA copy number variations. (A) Differentiation status of malignant cells analysed by CytoTRACE. (B) RNA velocity of malignant cells. (C) Differentiation trajectory of malignant cells, with each colour coded for clusters (left) and pseudo‐time (right). (D) Expression of FOSL1 in malignant cells was shown in the tSNE view. (E) The expression of EPCAM, TRIM29 and DKK1 was shown with violin plots. (F) Multiplex immunofluorescence staining of LC tissue. (G) Candidate drugs that target AREG and F3 in C3 or G6PD in C8 of malignant cells. (H) Bubble plots showed the expression of genes related to CIN was shown. (I) Expression of genes in CIN cells. (J) KEGG pathway of total epithelial cells enriched between PDL1 high compared with PDL1 low expression group. Circle means downregulated pathways and triangle means upregulated pathways. The dot represents the enriched gene number. (K) Expression of GATA6 was shown with violin plots and tSNE view. (L) Kaplan–Meier plots of overall survival (OS) for NSCLC patients with CTLs with the top and bottom TIDE prediction scores. The P value was calculated by testing the association between TIDE prediction scores and overall survival with the two‐sided Wald test in a Cox‐PH regression. (M) Expression of genes was shown with violin plots and tSNE view.

Cancer stem cells (CSCs) are a subpopulation of cells that contribute to cancer metastasis and resistance to chemotherapy or radiotherapy.^51^, ^52^ Therefore, we determined whether there were stem‐like clusters in malignant epithelial cells. CytoTRACE and RNA velocity analyses showed that Mep_C3 and C8 were less differentiated, while Mep_C6, C5, and C7 were highly differentiated (Figure 7A,B). Pseudo‐time analysis results also showed that Mep_C3 and C8 represented initiation points then branched in two directions (Figure 7C). Moreover, Mep_C3 and C8 were relatively quiescent and had lower metabolism (Figure S6D). These findings indicated that Mep_C3 and C8 contained CSCs. In addition, SCENIC analysis results showed that some stemness‐related transcription factors, such as FOSL1,^53^, ^54^ were obviously enriched in Mep_C3 and C8 (Figure 7D). we also evaluated the expression of some canonical CSC marker genes such as CD133,^55^ SOX2, NONOG, LGR5^56^ and found that they rarely expressed in the epithelial cell, but other genes that have been reported to regulate CSCs were also enriched in Mep_C3 and C8, such as HMGA1^57^ and PEG10^58^ (Figure S6E). To confirm Mep_C3 and _C8 presented in the LC tissue, we detected the main marker expression (EPCAM/DKK1/TRIM29) by applying multiplex immunohistochemistry and found that DKK1 and TRIM29 were specifically enriched in these cells (Figure 7E,F).

We calculated the number of potential targets of each subgroup with all the marker genes and determined candidate drugs and found that Mep_C9 had 31 potential drug targets; in contrast, only AREG and F3 were identified in Mep_C3, and G6PD was identified in Mep_C8 (Table S7). AREG, functioning as a promoter in epithelial malignancies,^59^, ^60^ is present in the TME and contributes to therapeutic resistance.^61^ F3 has been reported to play an important role in tumour growth, angiogenesis and metastasis.^62^, ^63^ G6PD activity is elevated in several types of cancer, including LC, and it promotes cancer growth and development by maintaining intracellular redox homeostasis.^64^ Because AREG, F3, and G6PD play a crucial role in tumour growth, angiogenesis and metastasis, the interruption of AREG by cetuximab, panitumumab, irinotecan, or capecitabine and of F3 by simvastatin or G6PD by trimethoprim might represent strategies to specifically kill CSCs and overcome chemoresistance in this cancer (Figure 7G).

In addition, we found that higher Mep_C3‐ and C8‐related gene signatures were associated with poorer survival in LUAD (Figure S6F). Specifically, high expression of FOSL1, DKK1 and TRIM29 was correlated with shorter survival (Figure S6G).

### 3.8 Epithelial cells are associated with chromosomal instability

IHC is the gold standard to distinguish tumour cells. Therefore, we performed IHC, and all the epithelial cells in the CNS were identified as tumour cells (Figure S7A). However, by adopting DNA CNV analysis, we identified 1788 epithelial cells with lower CNV (Figure S7B). As we know, chromosomal instability (CIN) consists of CNA in tumour cell chromosomes and plays multiple roles in cancer and its microenvironment.^65^, ^66^ To further understand the CIN‐associated feature of the cells between epithelial cells with higher‐ and lower‐CNV, we analysed the differential expressed genes and found a total of 60 downregulated genes and 118 upregulated genes were identified in the epithelial cells with higher CNV higher (Figure S7C). The results showed that higher‐CNV cells expressed genes correlated with higher CIN such as TMEM173,^67^ TP53/MDM2,^68^ EGFR,^69^ MACC1,^70^ FOS,^70^ UBAC2^71^ (Figure 7H). These results indicated that epithelial cells with higher‐ and lower‐CNV exhibited higher and lower CIN.

We further found that the cells with lower CIN had enrichment of ECM interaction and focal adhesion genes (Figure 7I). These results indicate that the lower CIN cells may play a role in cell seeding and colonisation and assist cells with higher CIN in metastasising into brain tissues; however, it needs to be further studied. The cells also showed activation of fructose and the mannose metabolism‐related genes However, the cells with higher CIN showed enrichment of genes that function in protein processing in the Endoplasmic Reticulum (ER) (Figure 7I). ER stress has been reported to endow malignant cells with greater tumourigenic, metastatic, drug‐resistant and immunosuppressive capacities.^72^ Our results indicate that cells with higher CIN had a stronger capability to adapt to ER stress.

### 3.9 Status of PDL1‐ or EGFR‐modified gene signatures of epithelial cells

As anti‐PD1/PDL1 and anti‐EGFR were the main effective anti‐immune therapy and TKIs targeted therapy, we further explored the gene expression alterations between PDL1 high and low expression, and EGFR mutation and wide type in all epithelial cells. The results showed that PDL1 higher epithelial cells were increased ECM–receptor interactions and the PI3K‐AKT signalling pathway while decreased antigen processing and presentation and protein processing in the ER than PDL1 lower cells (Figure 7J). SCENIC analysis showed that GATA6 was elevated in samples with higher PDL1 (Figure 7K). GATA6 has been reported to upregulate PDL1 expression in immortalised human sebocytes.^73^ We also found that the expression of GATA6 in the tumour cells elevated the infiltration of immune cells (Figure S7D) and the higher expression of GATA6 indicated the poor prognosis of higher infiltration of CTLs and indicated that these genes may involve in the dysfunction of CTLs (Figure 7L). Targeting GATA6 combined with an anti‐PDL1 antibody might provide an effective strategy for cancer therapy.

We next wondered whether EGFR mutation could determine the epithelial cell subpopulation and found that there was no significant difference in EGFR mutation status between cell clusters (Figure S7E). Next, we found that EGFR‐mutated cells showed activation of processes related to ribosomes, spliceosomes and oxidative phosphorylation pathways (Figure S7F). In addition, EGFR‐mutated cells showed downregulation of the transcription factors EST1 and FOSL1 and upregulation of EGR1 and HOXB9 (Figure 7M). Accumulating evidence has demonstrated that EGFR tyrosine kinase inhibitors (TKIs) evoke innate drug resistance by inactivating EST1 function.^74^, ^75^ EGFR mutation promotes the expression of EGR1,^76^, ^77^ and high early growth response 1 (EGR1) expression correlates with resistance to anti‐EGFR treatment.^78^ A higher level of DNA methylation of HOXB9 was found to be correlated with intrinsic EGFR‐TKI resistance and poor TKI response.^79^ Expression of mutant EGFR (EGFRvIII) resulted in upregulation of a small group of genes, including FOSL1,^80^ and EGFR‐PKM2 signalling induced the expression of FOSL1 to promote nasopharyngeal carcinoma cell invasion and metastasis.^81^ Our results will help to improve the response to EGFR‐TKI treatment.

## 4 DISCUSSION

Recent studies have found that TME significantly influenced therapeutic response and clinical outcome of cancer.^82^ Therefore, it will be beneficial to gain a deeper understanding of the TME composition. The unique TME in the brain, such as intracranial cell composition (including microglia, astrocytes and neurons), the blood–brain barrier (BBB), and the immunosuppression state, make it more complex than that in the extracranial tumours. Some recent reports showed the features of TME in LC without comparing with GM^48^, ^83^ or included TME comparison in brain metastasis and GM, but not at single cell level.^17^, ^84^ In our study, we applied single‐cell RNA‐seq to interrogate the TME landscape in GM and LC. We found that GM highly enriched immune‐activated mononuclear phagocyte populations macrophage M1 and microglia MG1 while LC increased the abundance of macrophages M2 and microglia MG2. Besides, GM showed enrichment endothelial cell cluster EC_C1 that increased immune‐related pathways, such as the TNF signalling pathway while LC‐enriched EC_C0 and C2 that activated angiogenesis and downregulated immune‐related pathways, such as the B cell receptor signalling pathway. Our analyses revealed major changes in TME cell populations, which were dependent on the tissue type of brain tumour.

The polarisation of TAMs in brain metastases is more complex than the classical M1/M2 model.^48^, ^85^ In our results, we clarified microglia into cytokine‐secreted MG1 (CX3CR1^+^/CCL3^+^/CCL4^+^) and MHCII highly expressed MG2 (CX3CR1^+^/HLA‐DRB1^+^/HLA‐DPA18). The polarisation process may dependent on metabolic pathways and pro‐inflammatory M1 TAM was reported to be supported by glycolysis, whereas anti‐inflammatory M2 TAM utilises fatty acid oxidation (FAO).^86^, ^87^ According to our results, M2 macrophages and MG2 microglia showed high activation of lipid metabolism processes. We also analysed TAM function according to the two functional states of macrophages (S100A8^+^/S100A9^+^/FCN1^+^/IL1B^+^ or APOE^+^/TREM2^+^/HLA‐DRA^high^) recently found by Gonzalez H in brain metastases^88^ and found that not all the TAM can be identified into these two function states. These further indicated the complex status of TAM. In our study, we also identified a subpopulation of proliferative macrophages whose gene signature was associated with a poor prognosis in LUAD. We also compared our results with the previous study that SPP1+ macrophages identified by Leaders^89^ in NSCLC primary foci and found that proliferative macrophages expressed SPP1. (Figure S8G) Targeting the proliferative TAM or inducing them to directly differentiate into cells with a tumour‐suppressive phenotype might be helpful for tumour immune therapy.

Due to the lack of expression of CD66B and LY6E, we could not sub‐classify neutrophils into canonical N1 and N2, but pseudo‐time analysis showed that clusters are two separate branches of the trajectory, suggesting that there may be two states of differentiation. Alternatively, we found a subset of ROS‐producing neutrophils that may be related to angiogenesis.^90^ We also validated the presence of ROS‐producing neutrophils in the public database URECA with the expression of S100A8^+^CSF3R^+^VEGFA^+^HIF1A^+^CXCL8^+^ in the lung brain metastasis tissue (Figure S8A–F). We also found that ROS‐producing neutrophils in bevacizumab (inhibitor of VEGF) treatment LC10 downregulated some pathways involved in adhesion and transendothelial migration which indicated that anti‐VEGF treatment such as bevacizumab may inhibit the angiogenesis and disrupt the communication of ECs and neutrophils (Figure S8I). Targeting ROS‐producing neutrophils with ROS inhibitors or ROS scavengers combined with VEGF inhibitors such as Bevacizumab may help to suppress tumour angiogenesis.

As the constituent part of blood–tumour interface (BTI), ECs played roles in angiogenesis and ECM remodelling or participant in immune‐related processes. Gonzalez et al.^88^ have revealed three endothelial clusters (EC‐1, EC‐2 and EC‐3) in brain metastases. Interestingly, EC_C0 in our results shared marker genes APLNR and ESM1 with their EC‐1, as well as biological processes such as angiogenesis and ECM remodelling; while C1 was partial of their venous‐like (EC‐2) which enriched more immune‐related processes, such as TNF signalling, antigen processing and presentation etc. Additionally, drug resistance‐related genes, including ABCB1 and ABCG2, were ubiquitously expressed in all ECs (Figure S8H). We also identified EC‐expressed genes that were associated with activation of ECM–receptor interactions and comprised a large portion of the cells interacting with fibroblasts; similar findings were reported in another previous study.^48^ Thus, ECs seem to be involved in a continuous process from immune cell infiltration to immune response and drug resistance in brain metastases BTI.

Considering the different outcomes of PD1 or PDL1 inhibitor clinical trials in GM and LC metastasis,^91^ we asked whether abundant immune cells in the tumour environment could alter therapeutic efficacy. In our study, we found that T cells were abundant in LC tissue than GM. CD8^+^ T Cell proportion directly determined the immune effect on tumour cells and the expression of PD1 inhibited the cytotoxic. In this study, CD8_TEX and proliferative CD8 T expressed PD1. To validate our result in other research, we sub‐cluster all the CD8+ cells into four metaclusters with the main marker identified by Sudmeier et al.^92^ The results showed that CD8_TEX and proliferative CD8 T cells expressed genes encoding co‐inhibitory molecules such as CTLA4, ENTPD1, HAVCR2, and LAG3; therefore, they can be defined by metacluster A and D (exhausted). CD8_Tem expressed TCF7 and IL7R while CD8_Temra expressed TCF7, IL7R and CD69 were included in metacluster B and C (memory‐like) (Figure S8J). These results demonstrated that CD8_TEX and proliferative CD8 T cells with distinct immune suppressive phenotypic in the TME. Consistently, our results showed that the abundance of proliferative CD8+ cells was elevated in the high PDL1 expression group and was associated with the downregulation immune checkpoint inhibition receptor genes LAG3, AHR3 and TIGIT. A combination of tumour immune therapy with an anti‐PD1/PDL1 inhibitor with other ICB inhibitors may improve the effectiveness of treatments for a brain tumour.

EGFR tyrosine kinase inhibitors treatment was the main strategy for patients diagnosed with lung cancer harbouring EGFR mutations, however, acquired drug resistance limited the treatment efficiency.^7^, ^8^, ^9^ Further understanding of the diversity of tumour cells with EGFR statuses may improve the clinical treatment. Our results showed that EGFR‐mutated cells regulated several transcription factors such as EST1 and FOSL1, EGR1 and HOXB9 which may be associated with the targeting effect of TKIs.^93^ Hypoxia in the tumour was reported to overcome TKIs drug resistance. Hypoxia can induce the expression of EGFR. On the other hand, EGFR might increase the expression of HIF‐1α to elevate the cellular response to hypoxia.^94^, ^95^ In our study, the expression of HIF‐1α and EGFR in epithelial cells was a positive correlation which was consistent with the previous study. The combination of the TKIs with an inhibitor of hypoxia‐inducible factor inhibitor acriflavine^96^ or hypoxia‐induced pathways such as FGFR1 inhibitors or MEK inhibitors^97^ was a promising strategy for patients with EGFR‐Mutant.

Since some studies showed that the EGFR activation pathway can increase the expression of PDL1,^98^ we wondered whether a combination TKIs with anti‐PD1/PDL1 drugs can improve the therapy efficiency. Therefore, we first compared the proportion of immune cells between the EGFR wild type and mutation group. It was very pitiful that we did not find any valuable cluster. Some studies reported that the combination therapy showed more beneficial efficacy than ICBs alone but increased treatment‐related adverse events except for patients with KRAS mutation.^99^ It was important to consider both safety and therapy efficacy when ICBs and TKIs are combined.

### 4.1 Limitations of this study

Due to the limitation of sample collection, we had not obtained gliomas in homogeneous tumour type or tumour driver genes. Though all of them were diagnosed with high‐grade glioma (WHO grade III–IV), the small sample number and heterogeneity of cancer subtype and driver mutations and the location in the brain may affect the results of different cell clusters between glioma and metastases. In addition, the history of treatment such as radiation or chemotherapy in lung brain metastasis may also affect the phenotypes and infiltrating of immune cells. More importantly, this study had several methodological limitations and was lack of functional experiments. More functional studies need to be further studied.

Overall, we show that the immune cell compartment of the brain TME is mainly shaped by LC metastases. Our results provide particular insights into the characteristics of microglia, macrophages, neutrophils, ECs and T cells. We also identified a subset of stem cell‐like cancer cells. Our study will deepen the understanding of the brain TME, thereby leading to improved patient management in precision medicine.

## CONFLICT OF INTEREST

The authors declare no conflict of interest.

## Supporting information

Supporting Information

## Data Availability

The data that support the findings of this study are available in the National Center for Biotechnology information Gene Expression Omibus (https://www.ncbi.nlm.nih.gov/geo/) with accession number GSE202371).
