# Supplementary material for: Single‐cell RNA sequencing reveals cellular and molecular reprograming landscape of gliomas and lung cancer brain metastases
Source: Clin Transl Med. 2022 Nov 6;12(11):e1101. doi: 10.1002/ctm2.1101 (PMC9637666; doi:10.1002/ctm2.1101)
Supplement: Supplementary file 1 — Supporting Information [file CTM2-12-e1101-s001.docx]

**Supplementary Figures**

**
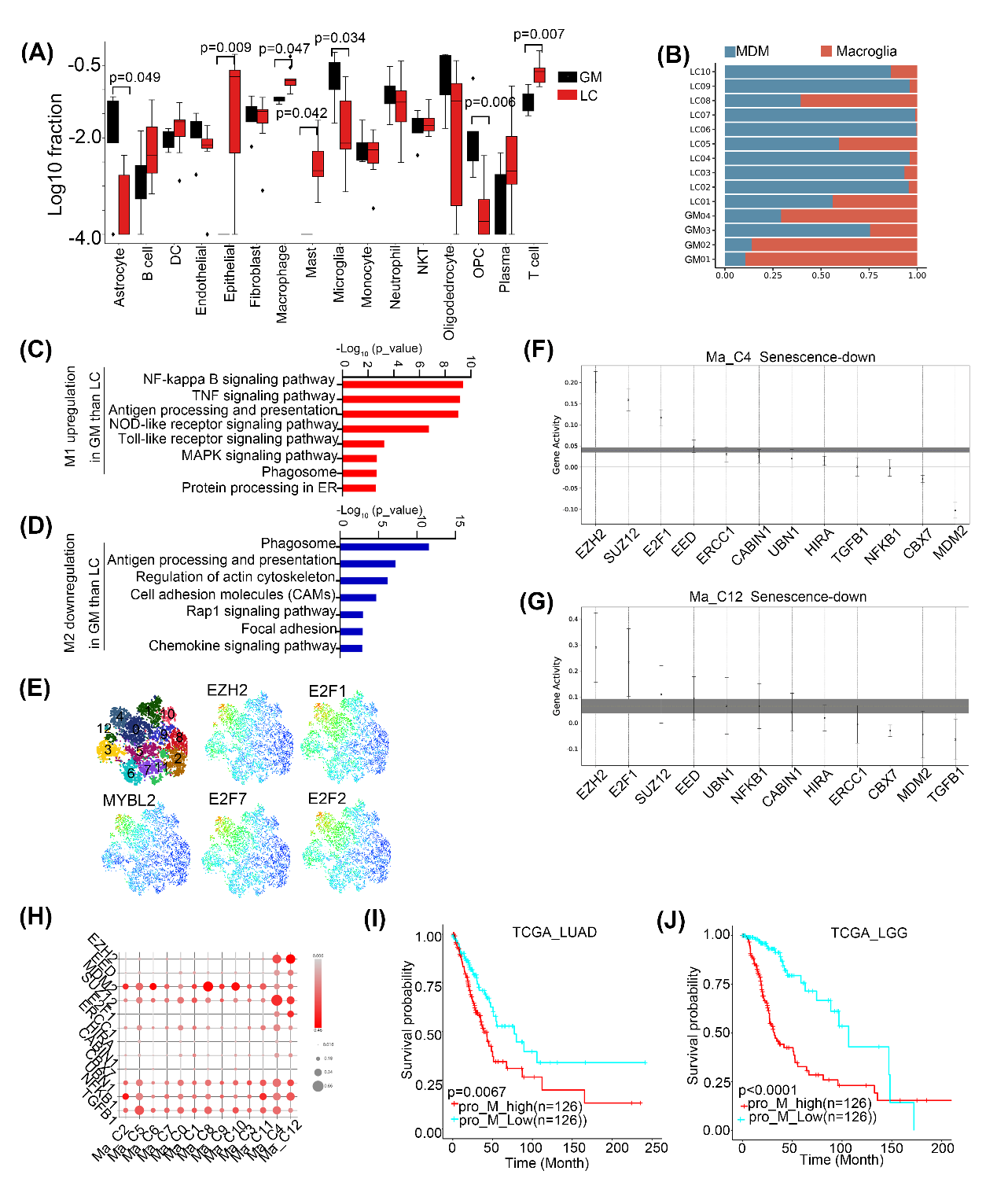
**

**Figure. S1. A subset of proliferative macrophage is associated with poor prognosis.**

(**A**) Cell type proportions in GM and LC, the black and red represented GM and LC, respectively. (**B**) Microglia and macrophage composition distribution for each patient sample. (**C**) KEGG pathway of M1 macrophage upregulated in GM than in LC. (**D**) KEGG pathway of M2 macrophage downregulated in GM than in LC. (**E**) The expression of indicated transcriptional factors of macrophage. (**F**) Gene activity of senescence-down related molecular in Ma_C4. (**G**) Gene activity of senescence-down related molecular in Ma_C12. (**H**) Gene bubble plot of senescence-down related genes. (**I**) The survival curve of proliferative macrophage gene signature in LUAD in TCGA. (**J**) The survival curve of proliferative macrophage gene signature in LGG in TCGA.

**
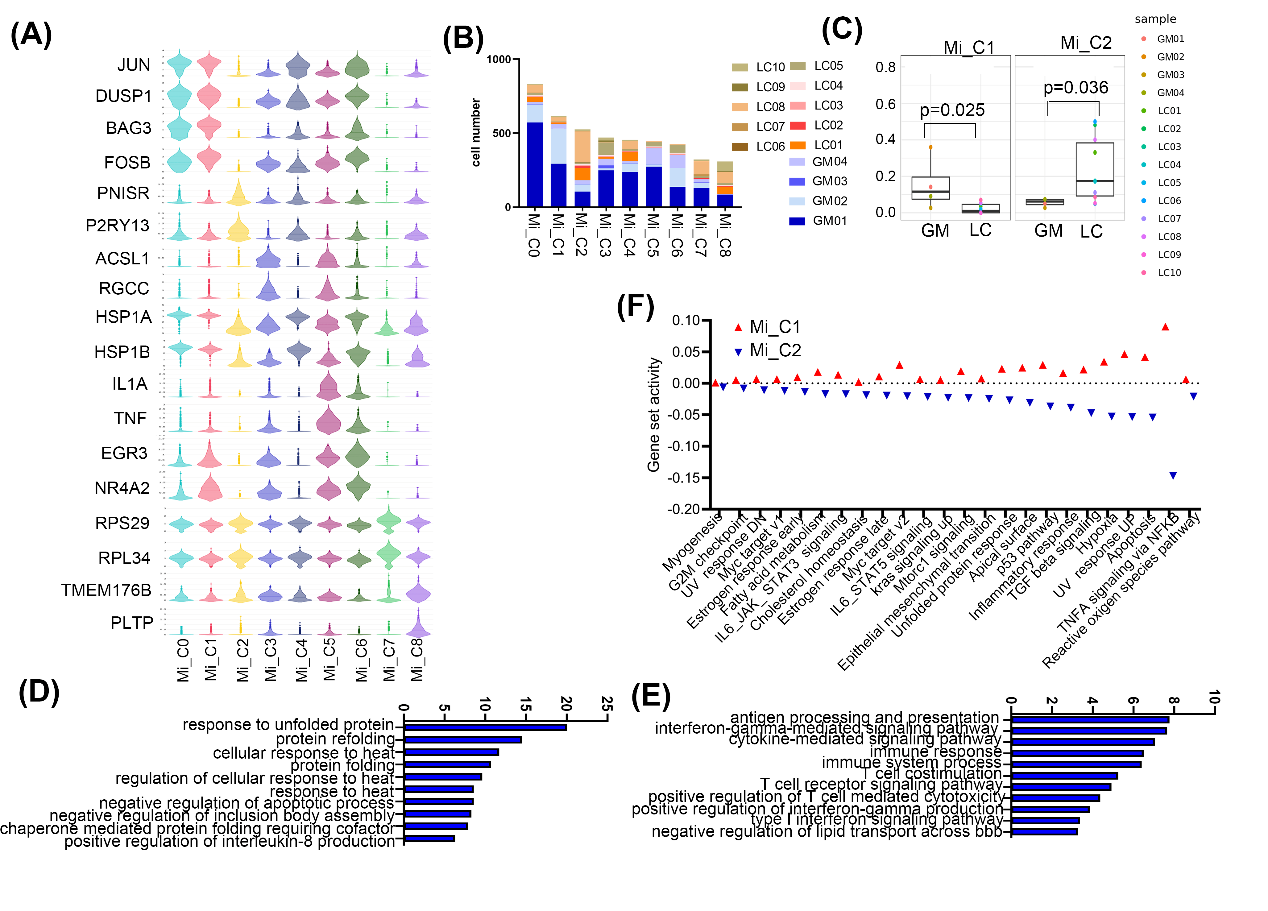
**

**Figure. S2. Microglia exhibited multiple polarization phenotypes in brain.**

(**A**) The violin plots of the marker gene expression in microglia clusters. (**B**) The relative proportion of different microglia clusters in GM and LC. (**C**) The relative proportion of C1 and C2 in GM and LC. (**D**) The upregulation of GO-BP (Gene Ontology Biological Processes) term enrichment in MG1 in GM compared with LC. (**E**) The upregulation of GO-BP (Gene Ontology Biological Processes) term enrichment in MG2 in LC compared with GM. (**F**) Gene set activity involves in pathway of Mi_C1 and Mi_C2 in GM compared with LC.


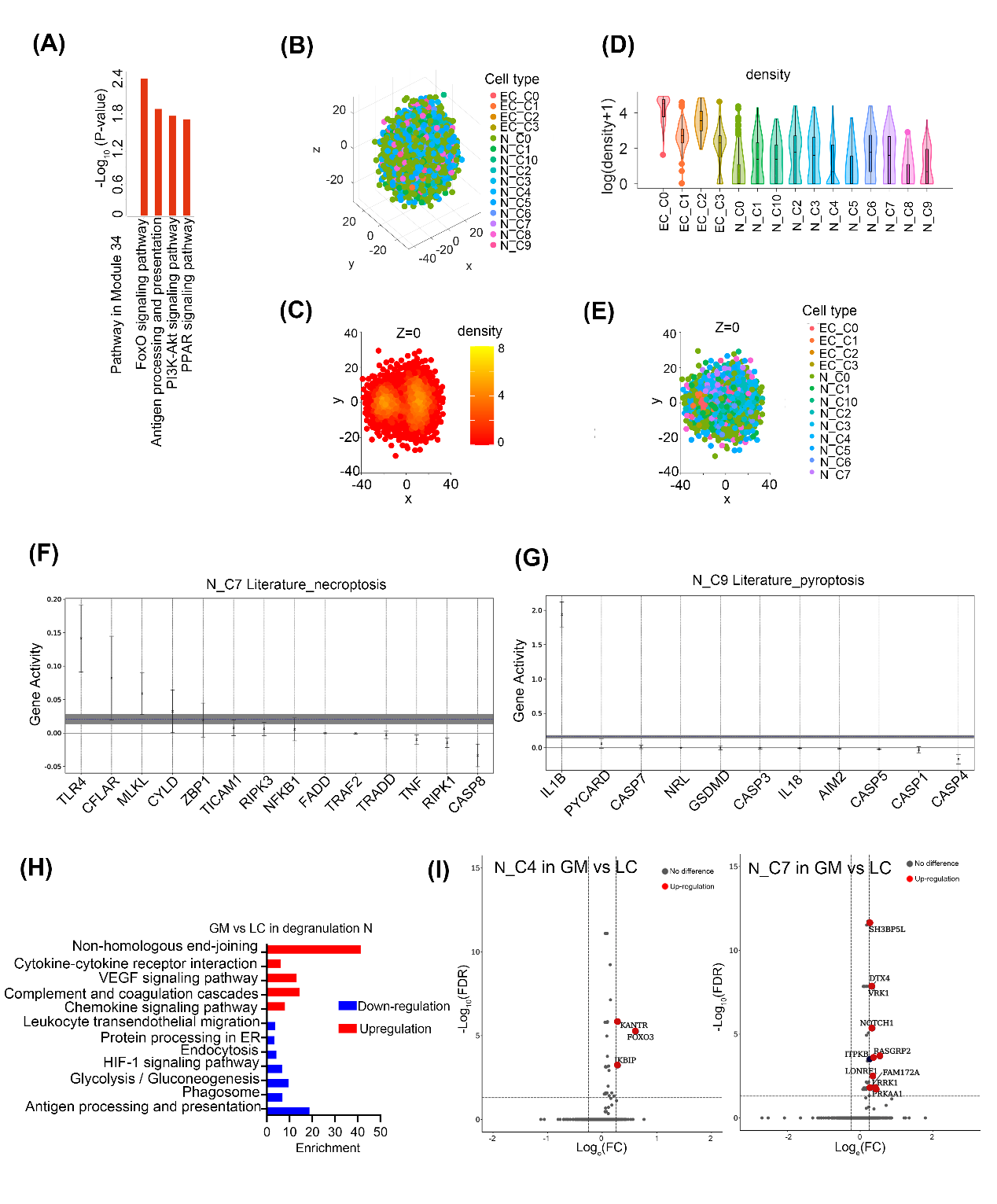


**Figure. S3. ROS productive neutrophils may participate in tumor angiogenesis in brain malignancies.**

(**A**) Pathways enriched in module 34 in neutrophils. (**B**) Spatial organization of neutrophils and endothelial cells in the pseudo-space inferred by CSOmap based on the scRNA-seq data. Each dot represents a cell, and its color represents the corresponding

cell cluster. (**C**) The cross-section of z = 0 of the pseudo-space. The color of the dots represents cell density. (**D**)The difference in cell density between neutrophils and endothelial cell clusters. (**E**) Location of neutrophil cell cluster and endothelial cell clusters in the cross-section of pseudo-space z = 0. (**F**) Gene activity of necroptosis related molecular in N_C7. (**G**) Gene activity of necroptosis related molecular in N_C9. (**H**) KEGG pathway enriched in degranulated neutrophils in GM compared with LC. Red color represents upregulation and blue color means downregulation. (**I**) The volcano plot of variable expression genes of N_C4 and C7 neutrophils between GM verse LC.

**
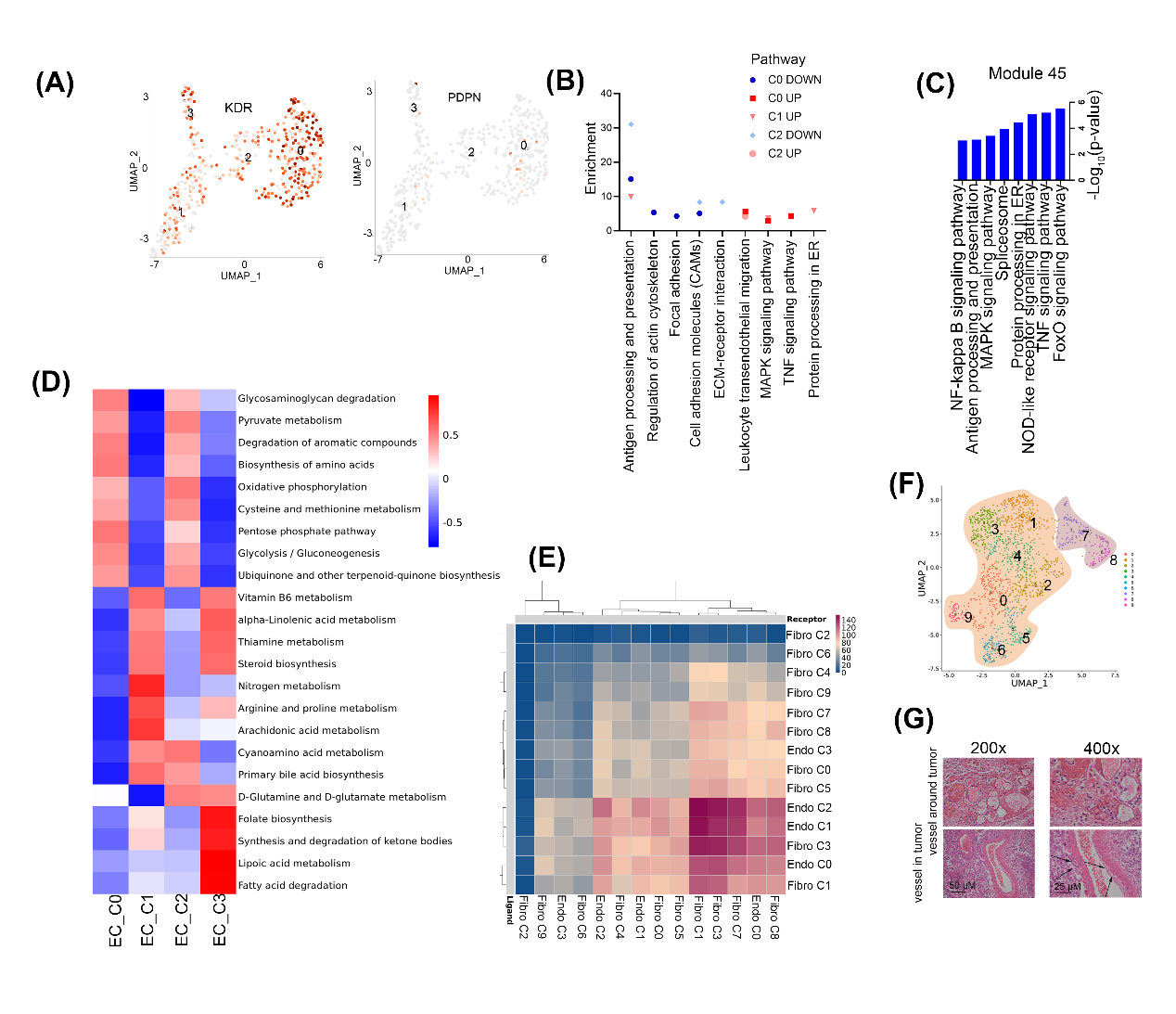
Figure. S4. Endothelial cells communicate with tumor associated fibroblast cells**

(**A**) Expression of KDR and PDPN in ECs was showed in UMAP view. (**B**) KEGG pathway enriched in EC_C0, C1 and C2 in GM compared with LC. Red color represents upregulation and blue color means downregulation. (**C**) Pathway enriched in module 45 in ECs. (**D**) Metabolism pathway enriched in EC clusters. Red color represents upregulation and blue color means downregulation. (**E**) Heatmap of interactions between EC clusters with fibroblast clusters. (**F**) The UMAP view of fibroblast. (**G**) The H&E staining in LC07.


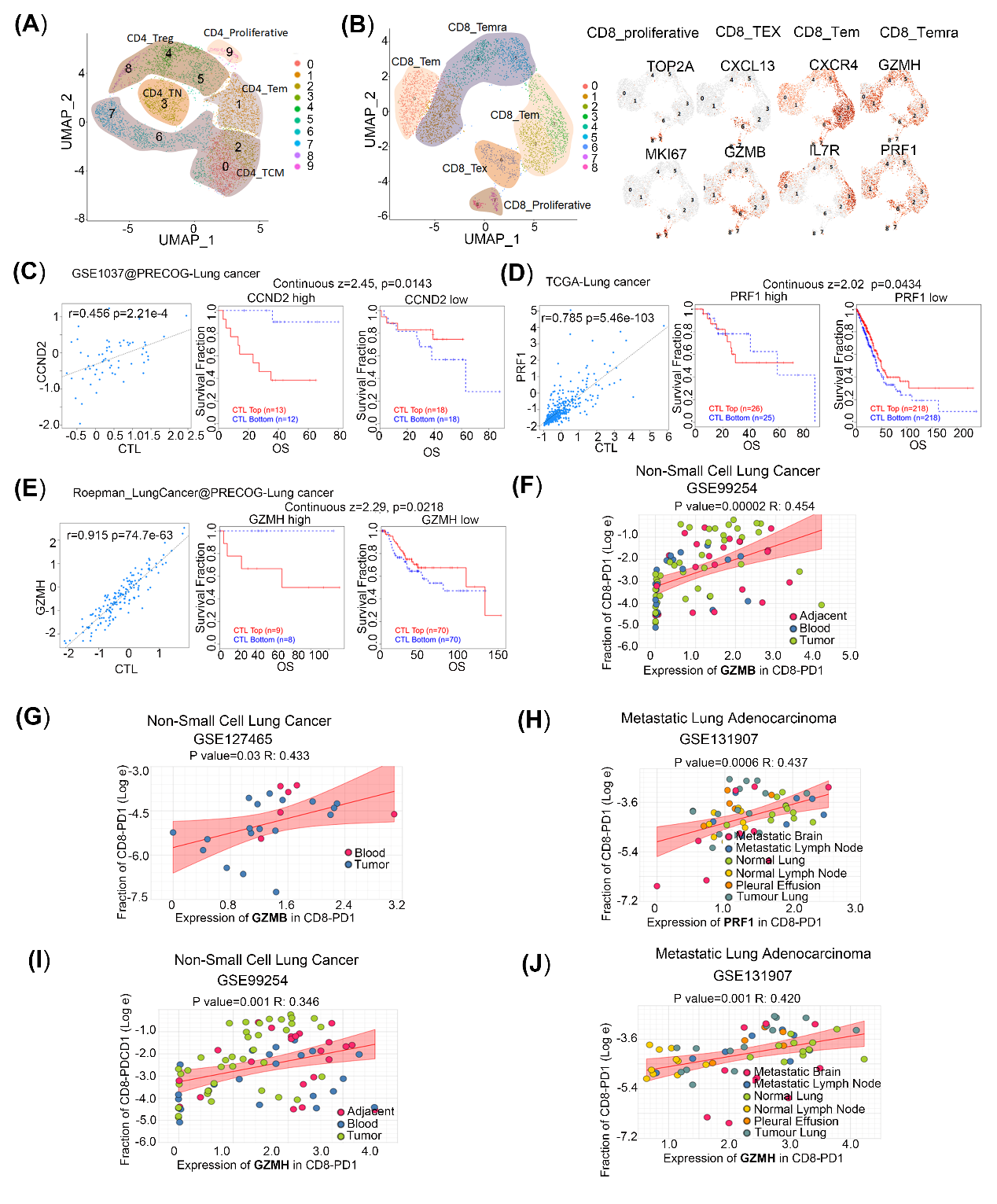


**Figure. S5. The heterogeneity for T cells and the association with the expression of PDL1**

**(A**) The UMAP view of different clusters of CD4^+^ T cells. (**B**) The UMAP view (the right panel) and the main marker gene expression of subtype of CD8^+^ T cells (the left panel). (**C-E**) Correlation between the gene expression of CCND2, PRF1, GZMH and the infiltration of CTLs was showed on the left and the Kaplan–Meier plots of overall survival (OS) for LUAD patients with CTLs with the top and bottom TIDE prediction scores based on gene expression of CCND2, PRF1, and GZMH (on the right). The P value was calculated by testing the association between TIDE prediction scores and overall survival with the two-sided Wald test in a Cox-PH regression. (**F-J**) Correlation of expression of PRF1, GZMH in CD8^+^ PD1^+^ cells with the fraction of CD8^+^ PD1^+^ cells in metastatic LUAD or NSCLC.


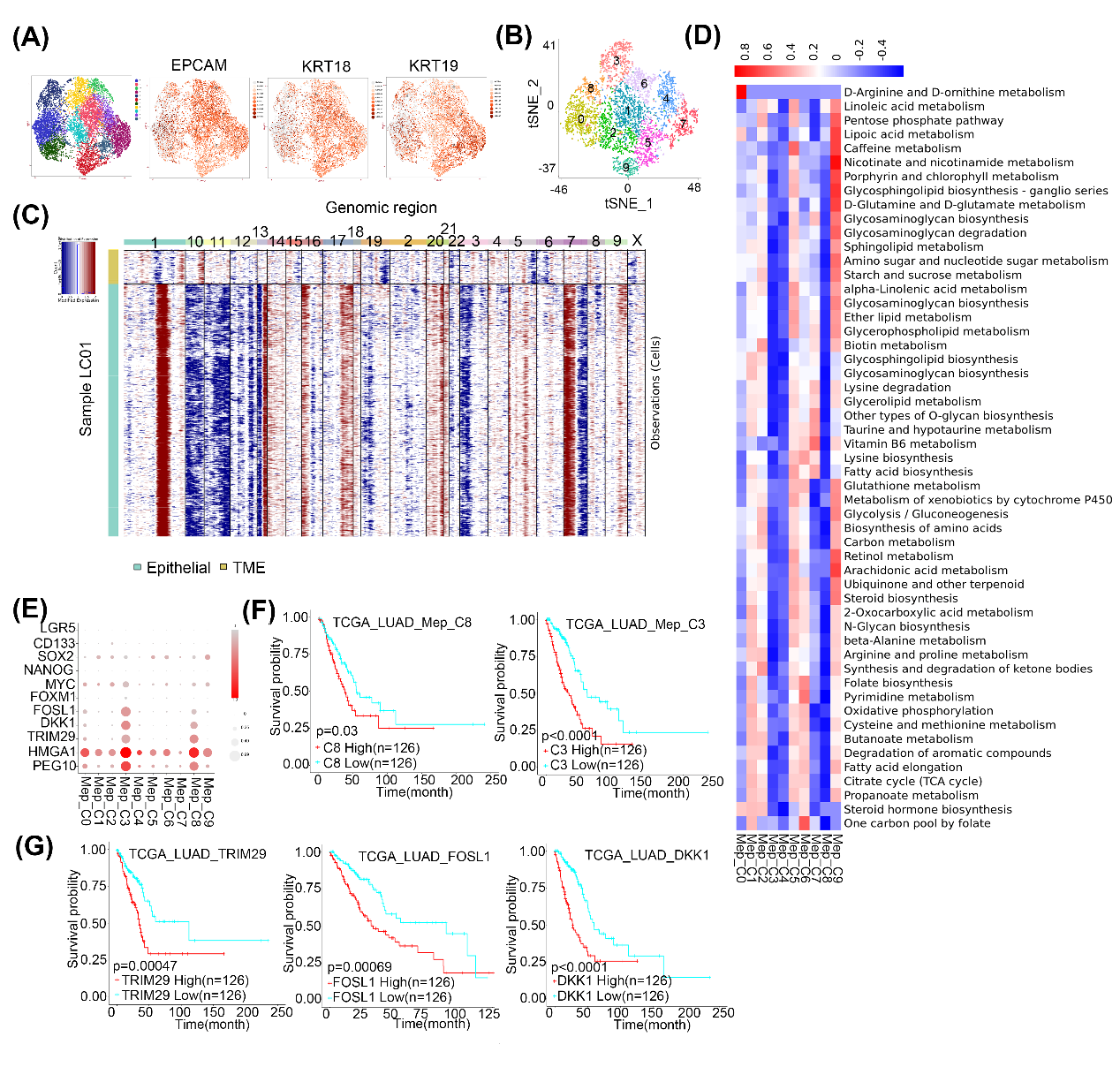


**Figure. S6. Epithelial cells harbor a subset of cancer stem cells and show heterogeneity of DNA copy number variations**

(**A**) The UMAP view of total epithelial cells and the expression of EPCAM, KRT18 and KRT19. (**B**) The tSNE view of malignant cells. (**C**) Inferred CNA for cells in LC01. (**D**) Metabolism pathway enriched in CNV higher cells. Red color represents upregulation and blue color means downregulation. (**E**) Bubble plots showed the expression of CSC related genes in malignant epithelial cell clusters (Mep_C). (**F**) The survival curve of Mep_C8 and Mep_C3 in CNV higher cells gene signature in LUAD in TCGA. (**G**) The survival curve of expression of TRIM29, FOSL1 and DKK1 in LUAD in TCGA, respectively.


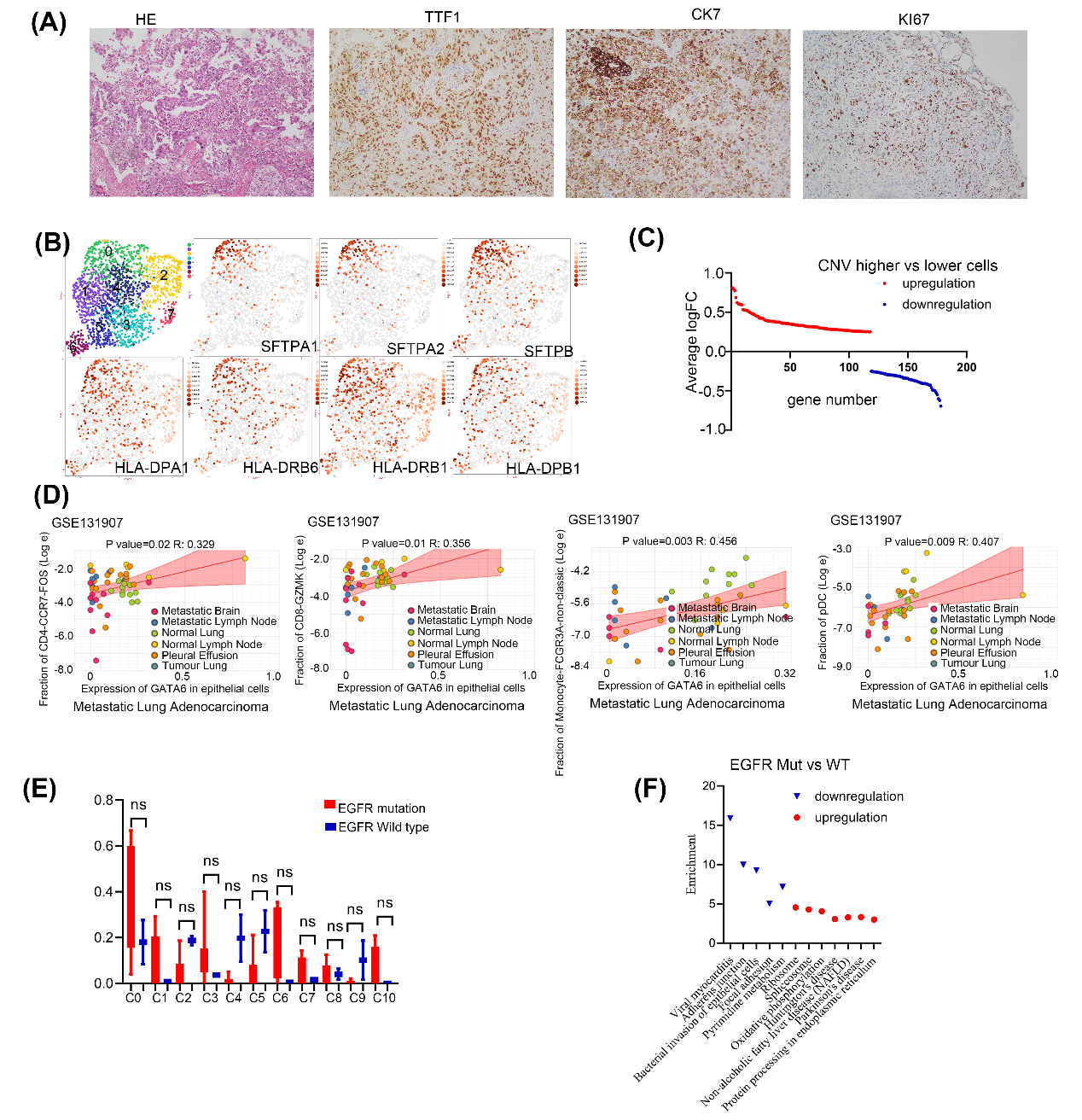


**Figure. S7. Status of PDL1- or EGFR-modified gene signatures of epithelial cells**

(**A**) HE staining in sample LC07 and the expression of TTF1, CK7 and KI67 analyzed by IHC. (**B**) Expression of genes in CNV lower cells was showed in UMAP views.

(**C**) The different expression genes in higher CNV cells compared with lower CNV cells. Red color represents upregulation and blue color means downregulation.

(**D**)Correlation of expression of GATA6 in epithelial cells with the fraction of different type of immune cells (CD4-CCR7-FOS, CD8-GZMK, Monocyte-FCGR3A and pDC) in metastatic LUAD based on GSE131907. (**E**) Cell proportion of epithelial cells in EGFR mutation and wild type group. (**F**) Pathway enriched in EGFR-mutated compared with EGFR wild type, red color represents upregulation and blue color means downregulation.


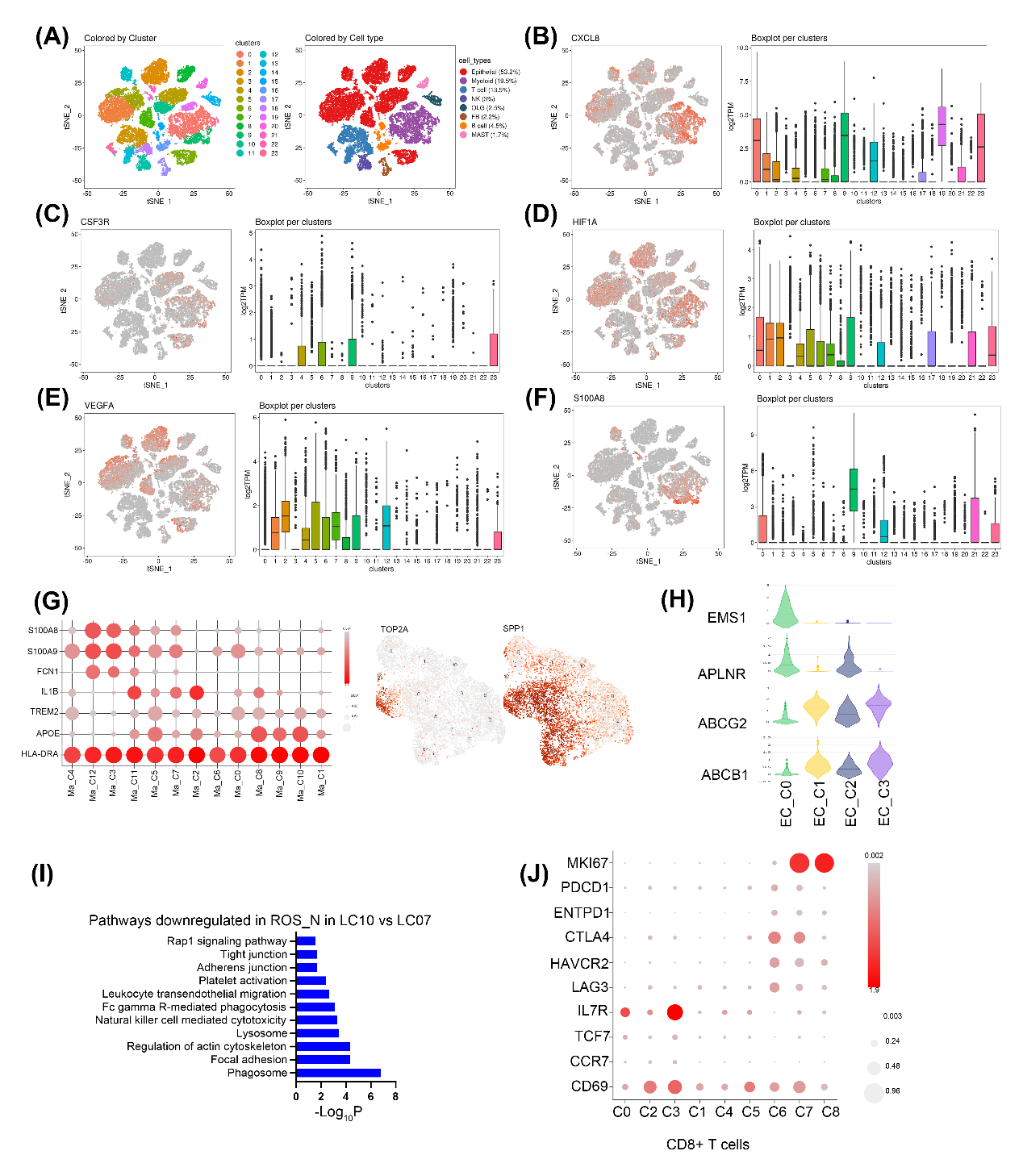


**Figure S8 Validation the results in this study in the published datasets or public database.**

(**A**) The cell cluster and cell type were showed in tSNE view. (**B**) -(**F**) the expression of marker genes in ROS productive neutrophils (CXCL8, CSF3R, HIF1A, VEGFA and S100A8) were shown in tSNE view (the left panel) and boxplots (the right panel). (**G**) Bubble plot showed the expression of marker genes of the two functional states of macrophages found by Gonzalez H in brain metastases (the left panel); the tSNE view showed the expression of TOP2A and SPP1 in our MDMs cell clusters(the right panel). (**H**) Violin plots showed the marker expression of the three endothelial clusters (EC-1, EC-2, and EC-3) identified by Gonzalez et al. (**I**) The pathways downregulated in ROS-producing neutrophils in LC10 compared with LC07. (**J**) Subcluster into Bubble plots showed marker genes expression involved in four metaclusters in all the CD8+ cell identified in this study.

**Supplementary Tables:**

**Supplementary Table 1. Clinical features and patient assignment per analysis.**

| **ID** | **Diagnosis** | **IDH1 R132H** | **MGMT** | **EGFR** | **PD-L1** | **Sex** | **Age** | **Location/side** | **Therapy prior surgery** | **Smoking** |
| --- | --- | --- | --- | --- | --- | --- | --- | --- | --- | --- |
| **GM01** | Glioblastoma | WT | Methylated |  |  | M | 47 | Temporal / Left | no | no |
| **GM02** | Anaplastic Oligodendroglioma | Mut | Methylated |  |  | F | 54 | Frontal / Left | no | no |
| **GM03** | Oligodendrogliom and Astrocytoma | Mut | Methylated |  |  | M | 49 | Frontal / Left | no | yes |
| **GM04** | Glioblastoma | WT | Methylated |  |  | F | 76 | Frontal / Right | no | no |
| **LC01** | Lung adenocarcinoma |  |  | Exon19 Mut | miss | F | 51 | Frontal / Right | no | no |
| **LC02** | Lung adenocarcinoma |  |  | Exon19 Mut | <1% | F | 70 | Frontal / Right | 4 years after lung cancer surgery, no chemoradiation; newly diagnosis with brain metastasis | no |
| **LC03** | Lung adenocarcinoma |  |  | WT | 20% | F | 64 | Occipital / Right | RT 30Gy - 4 x AC; 4 x Docetaxel; PD | no |
| **LC04** | Lung adenocarcinoma |  |  | Exon21 Mut | 1% | M | 66 | Parietal / Right | no | no |
| **LC05** | Lung adenocarcinoma |  |  | Exon19 Mut | 20% | F | 44 | Frontal / Left | no | no |
| **LC06** | Lung adenocarcinoma |  |  | Exon20 Mut | <1% | F | 46 | Parietal / Right | no | No |
| Continued | | | | | | | | | | |
| **ID** | **Diagnosis** | **IDH1 R132H** | **MGMT** | **EGFR** | **PD-L1** | **Sex** | **Age** | **Location/side** | **Therapy prior surgery** | **Smoking** |
| **LC07** | Lung adenocarcinoma |  |  | Exon21 Mut | 20% | F | 52 | Frontal / Left | RT 30Gy - Icotinib;Xeloda; PD | no |
| **LC08** | Lung adenocarcinoma |  |  | Exon19 Mut | no expression | F | 61 | Frontal / Left | Gefitinib; PD | no |
| **LC09** | Lung adenocarcinoma |  |  | WT | >80% | M | 58 | Parietal / Left | no | yes |
| **LC10** | Lung adenocarcinoma |  |  | WT | miss | F | 50 | Frontal / Right | RT 30Gy - 4 x AC; Erlotinib; 4 x Bevacizumab+pemetrexed; PD | no |

Footnotes:

a, Mut: mutation

b, WT: wild type

c, RT: Radiotherapy

d, AC: Pemetrexed + Carboplatin

e, PD: Progressive disease

f, M: Male

g, F: Female

**Supplementary Table 2. Clinical features and Molecular pathology of patient.**

| ID | Diagnosis | WHO Grade | Molecular pathology |
| --- | --- | --- | --- |
| GM01 | Glioblastoma | Ⅳ | IDH mutation（-），MGMT promoter methylation（+），TERT（+），BRAF（-） |
| GM02 | Anaplastic Oligodendroglioma | III | IDH mutation（+），MGMT promoter methylation（+），TERT（+），BRAF（-） |
| GM03 | Anaplastic Oligodendrogliom and Astrocytoma | III | IDH mutation（+），MGMT promoter methylation（+），TERT（+），BRAF（-） |
| GM04 | Glioblastoma | Ⅳ | IDH mutation（-），MGMT promoter methylation（+），TERT（-），BRAF（-） |
| LC01 | Lung adenocarcinoma |  | EGFR-Exon19（+）；ALK（-） |
| LC02 | Lung adenocarcinoma |  | EGFR-Exon19（+）；ALK（-），MET（-），ROS1（-） |
| LC03 | Lung adenocarcinoma |  | EGFR（-）；ALK（-），MET（-），ROS1（-） |
| LC04 | Lung adenocarcinoma |  | EGFR-Exon21（+）；ALK（-），MET（-），ROS1（-） |
| LC05 | Lung adenocarcinoma |  | EGFR-Exon19（+）；ALK（-），MET（-），ROS1（-） |
| LC06 | Lung adenocarcinoma |  | EGFR-Exon20（+）；ALK（-），MET（+），ROS1（-） |
| LC07 | Lung adenocarcinoma |  | EGFR-Exon21（+）；ALK（-），MET（+），ROS1（-） |
| LC08 | Lung adenocarcinoma |  | EGFR-Exon19（+）；ALK（-），MET（-），ROS1（-） |
| LC09 | Lung adenocarcinoma |  | EGFR（-）；ALK（-），MET（+），ROS1（-） |
| LC10 | Lung adenocarcinoma |  | EGFR（-）；ALK（-），MET（-），ROS1（-） |

**Supplementary Table 3. The data quality of scRNA-seq per sample.**

| #Sample | RawReads(M) | CleanReads(M) | Cellidentified | ReadsCell | MedianGenes | MedianUMI | FilterdCell | AveragerUMI | AveragerGene | AveragerMito |
| --- | --- | --- | --- | --- | --- | --- | --- | --- | --- | --- |
| GM01 | 572.9822044 | 529.9692307 | 5469 | 28163 | 1728 | 4660 | 5143 | 5880.952557 | 1913.929419 | 0.145447138 |
| GM02 | 701.5286598 | 647.7827091 | 3824 | 47328 | 637.5 | 1771 | 2701 | 3734.338393 | 1264.632358 | 0.117762568 |
| GM03 | 951.1415653 | 887.2921352 | 5551 | 38082 | 2330 | 4806 | 4711 | 6737.947145 | 2668.188283 | 0.188171609 |
| GM04 | 674.8469925 | 601.8522663 | 5141 | 35076 | 1585 | 3508 | 4139 | 5743.234598 | 2115.780865 | 0.186347964 |
| LC01 | 1137.316257 | 1049.643431 | 5537 | 55068 | 2128 | 4814 | 4686 | 6598.155356 | 2469.765472 | 0.162608038 |
| LC02 | 1151.045475 | 1066.71781 | 5196 | 62620 | 944 | 1926.5 | 4999 | 4029.367073 | 1453.033007 | 0.141047659 |
| LC03 | 660.5085182 | 615.9091797 | 5414 | 36144 | 1581.5 | 3515.5 | 5278 | 9036.954149 | 2395.909814 | 0.118776048 |
| LC04 | 719.4125023 | 639.3388119 | 5586 | 35403 | 1361 | 2757.5 | 5223 | 6196.850469 | 2026.960559 | 0.150379996 |
| LC05 | 587.3617744 | 546.2445869 | 4891 | 33951 | 1004 | 1895 | 3975 | 6015.80956 | 1936.340629 | 0.12882571 |
| LC06 | 810.9628601 | 710.4712009 | 5077 | 40806 | 959 | 1790 | 4203 | 5532.033547 | 1748.956222 | 0.089092062 |
| LC07 | 1052.544312 | 963.7439537 | 5224 | 52788 | 1492.5 | 2985 | 4491 | 7313.194166 | 2277.419506 | 0.084495287 |
| LC08 | 722.6871624 | 651.8784714 | 5259 | 34055 | 1075 | 2268 | 4516 | 4235.015722 | 1541.54628 | 0.115477675 |
| LC09 | 801.773283 | 756.2180958 | 4452 | 50968 | 731 | 1458.5 | 4056 | 6134.245809 | 1748.286982 | 0.111246943 |
| LC10 | 831.0048885 | 779.9594383 | 3791 | 58856 | 1359 | 3128 | 3746 | 4595.427656 | 1592.398025 | 0.094081484 |

**Supplemental table 4 The gene list of macrophage M1 and M2 scoring.**

| Gene.name | GeneSet Name | Gene.name | GeneSet Name |
| --- | --- | --- | --- |
| CXCR4 | M2_Gene | HSD11B1 | M1_Gene |
| HNMT | M2_Gene | ATF3 | M1_Gene |
| CD302 | M2_Gene | CCL20 | M1_Gene |
| SLC4A7 | M2_Gene | CXCL9 | M1_Gene |
| HEXB | M2_Gene | CXCL11 | M1_Gene |
| TGFBI | M2_Gene | IL15 | M1_Gene |
| CHN2 | M2_Gene | IRF1 | M1_Gene |
| MSR1 | M2_Gene | EDN1 | M1_Gene |
| CA2 | M2_Gene | TNF | M1_Gene |
| MS4A6A | M2_Gene | PDGFA | M1_Gene |
| MS4A4A | M2_Gene | IL6 | M1_Gene |
| SLCO2B1 | M2_Gene | INHBA | M1_Gene |
| MRC1 | M2_Gene | NAMPT | M1_Gene |
| EGR2 | M2_Gene | IDO1 | M1_Gene |
| LIPA | M2_Gene | GADD45G | M1_Gene |
| CLEC7A | M2_Gene | SLC2A6 | M1_Gene |
| LTA4H | M2_Gene | PFKP | M1_Gene |
| LPAR6 | M2_Gene | IL2RA | M1_Gene |
| SLC38A6 | M2_Gene | PFKFB3 | M1_Gene |
| HS3ST2 | M2_Gene | OAS2 | M1_Gene |
| CCL13 | M2_Gene | BCL2A1 | M1_Gene |
| CCL23 | M2_Gene | SLC7A5 | M1_Gene |
| CCL18 | M2_Gene | CCL5 | M1_Gene |
| TPST2 | M2_Gene | IGFBP4 | M1_Gene |
| CERK | M2_Gene | CCR7 | M1_Gene |
| TGFBR2 | M2_Gene | SPHK1 | M1_Gene |
| IGF1 | M2_Gene | APOL6 | M1_Gene |
| FGL2 | M2_Gene | APOL3 | M1_Gene |
| P2RY13 | M2_Gene | APOL2 | M1_Gene |
| CLEC10A | M2_Gene | APOL1 | M1_Gene |
| ADK | M2_Gene | TYMP | M1_Gene |
| MAF | M2_Gene | IRF7 | M1_Gene |
| ALOX15 | M2_Gene | CXCL10 | M1_Gene |
| HS3ST1 | M2_Gene | TNFSF10 | M1_Gene |
| TLR5 | M2_Gene | SLC31A2 | M1_Gene |
| HRH1 | M2_Gene | OASL | M1_Gene |
| GAS7 | M2_Gene | PSME2 | M1_Gene |
| P2RY14 | M2_Gene | PLA1A | M1_Gene |
| Continued | | | |
| Gene.name | GeneSet Name | Gene.name | GeneSet Name |
|  |  | PSMA2 | M1_Gene |
|  |  | PSMB9 | M1_Gene |
|  |  | XAF1 | M1_Gene |
|  |  | BIRC3 | M1_Gene |
|  |  | PTX3 | M1_Gene |
|  |  | SLCO5A1 | M1_Gene |
|  |  | IL7R | M1_Gene |
|  |  | CHI3L2 | M1_Gene |
|  |  | IL12B | M1_Gene |
|  |  | VCAN | M1_Gene |
|  |  | FAS | M1_Gene |
|  |  | AK3 | M1_Gene |
|  |  | HESX1 | M1_Gene |
|  |  | IL15RA | M1_Gene |
|  |  | CCL19 | M1_Gene |
|  |  | CCL15 | M1_Gene |

**Supplemental table 5 The prognosis of TAM of GMB in TCGA.**

| Type | TCGA-GBM-high.Num | TCGA-GBM-low.Num | TCGA-GBM-high.MeanExp | TCGA-GBM-low.MeanExp | pvalue |
| --- | --- | --- | --- | --- | --- |
| M1 | 40 | 40 | 0.636722 | -0.44575 | 0.161503 |
| M_MDSC | 40 | 40 | 0.542281 | -0.36015 | 0.081854 |
| M2 | 40 | 40 | 0.795578 | -0.39785 | 0.157207 |
| Proliferative_Macrophage | 40 | 40 | 0.961186 | -0.75085 | 0.720115 |

**Supplemental table 6 The prognosis of microglia of GMB in TCGA.**

| Microglia cluster | TCGA-GBM-high.Num | TCGA-GBM-low.Num | TCGA-GBM-high.MeanExp | TCGA-GBM-low.MeanExp | pvalue |
| --- | --- | --- | --- | --- | --- |
| 0 | 40 | 40 | 0.996856343 | -0.557663636 | 0.229394 |
| 5 | 40 | 40 | 0.837193223 | -0.385414865 | 0.810318 |
| 6 | 40 | 40 | 0.875234059 | -0.430130091 | 0.435896 |
| MG1 | 40 | 40 | 20.8261966725153 | 6.72038408399812 | 0.284712943702479 |

**Supplemental table 7 The drug target and candidate drug in CNV higher epithelial cells.**

| cluters | allmarkers.gene | drugName |
| --- | --- | --- |
| Cluster7 | ABCC3 | Morphine |
| Cluster7 | ABCC3 | Vincristine |
| Cluster7 | ABCC3 | Doxorubicin |
| Cluster7 | ABCC3 | Cyclophosphamide |
| Cluster7 | ABCC3 | Methotrexate |
| Cluster7 | ABCC3 | Cisplatin |
| Cluster5 | ACHE | Rivastigmine |
| Cluster0 | ACTG1 | Vincristine |
| Cluster9 | ADM | Paroxetine |
| Cluster9 | APOC1 | Ritonavir |
| Cluster0 | AREG | Irinotecan |
| Cluster0 | AREG | Panitumumab |
| Cluster0 | AREG | Capecitabine |
| Cluster0 | AREG | Cetuximab |
| Cluster1 | ATP5E | Adalimumab |
| Cluster1 | ATP5E | Certolizumab pegol |
| Cluster1 | ATP5E | Infliximab |
| Cluster1 | ATP5E | Etanercept |
| Cluster1 | ATP5E | Methotrexate |
| Cluster2 | C3 | Clozapine |
| Cluster7 | CA12 | Zonisamide |
| Cluster7 | CA12 | Topiramate |
| Cluster9 | CALU | Warfarin |
| Cluster9 | CALU | Acenocoumarol |
| Cluster7 | CAMK1D | Losartan |
| Cluster9 | CAPG | Vincristine |
| Cluster9 | CCND1 | Methotrexate |
| Cluster9 | CCND1 | Fluorouracil |
| Cluster9 | CCND1 | Cetuximab |
| Cluster2 | CFH | Ranibizumab |
| Cluster2 | CFH | Bevacizumab |
| Cluster2 | COL1A1 | Somatotropin |
| Cluster2 | COL1A1 | Valproic Acid |
| Cluster1 | COMT | Sumatriptan |
| Cluster1 | COMT | Sufentanil |
| Cluster1 | COMT | Methadone |
| Cluster1 | COMT | Fluvoxamine |
| Cluster1 | COMT | Paroxetine |
| Cluster1 | COMT | Entacapone |
| Continued | | |
| cluters | allmarkers.gene | drugName |
| Cluster1 | COMT | Haloperidol |
| Cluster1 | COMT | Nicotine |
| Cluster1 | COMT | Oxycodone |
| Cluster1 | COMT | Morphine |
| Cluster1 | COMT | Clozapine |
| Cluster1 | COMT | Quetiapine |
| Cluster1 | COMT | Tramadol |
| Cluster1 | COMT | Bupropion |
| Cluster1 | COMT | Venlafaxine |
| Cluster1 | COMT | Cisplatin |
| Cluster1 | COMT | Risperidone |
| Cluster1 | CXCL8 | Sunitinib |
| Cluster1 | CXCL8 | Bevacizumab |
| Cluster1 | DUSP1 | Salbutamol |
| Cluster7 | EGFR | Ribavirin |
| Cluster7 | EGFR | Peginterferon alfa-2b |
| Cluster7 | EGFR | Irinotecan |
| Cluster7 | EGFR | Peginterferon alfa-2a |
| Cluster7 | EGFR | Panitumumab |
| Cluster7 | EGFR | Carboplatin |
| Cluster7 | EGFR | Fluorouracil |
| Cluster7 | EGFR | Tegafur |
| Cluster7 | EGFR | Erlotinib |
| Cluster7 | EGFR | Gefitinib |
| Cluster7 | EGFR | Imatinib |
| Cluster7 | EGFR | Leucovorin |
| Cluster7 | EGFR | Gemcitabine |
| Cluster7 | EGFR | Cetuximab |
| Cluster7 | EGFR | Docetaxel |
| Cluster7 | EGFR | Paclitaxel |
| Cluster9 | EPHX1 | Carbamazepine |
| Cluster9 | EPHX1 | Warfarin |
| Cluster9 | EPHX1 | Phenprocoumon |
| Cluster9 | EPHX1 | Cyclophosphamide |
| Cluster9 | EPHX1 | Phenytoin |
| Cluster9 | EPHX1 | Cisplatin |
| Cluster9 | EPHX1 | Docetaxel |
| Cluster3 | F3 | Simvastatin |
| Cluster5 | FCER1G | Acetylsalicylic acid |
| Cluster8 | G6PD | Dapsone |
| Cluster8 | G6PD | Nitrofurantoin |
| Cluster8 | G6PD | Amodiaquine |
| Continued | | |
| cluters | allmarkers.gene | drugName |
| Cluster8 | G6PD | Chloroquine |
| Cluster8 | G6PD | Primaquine |
| Cluster8 | G6PD | Acetylsalicylic acid |
| Cluster8 | G6PD | Sulfametopyrazine |
| Cluster8 | G6PD | Sulfasalazine |
| Cluster8 | G6PD | Sulfamethoxazole |
| Cluster8 | G6PD | Sulfadoxine |
| Cluster8 | G6PD | Ciprofloxacin |
| Cluster8 | G6PD | Chloramphenicol |
| Cluster8 | G6PD | Trimethoprim |
| Cluster8 | G6PD | Pyrimethamine |
| Cluster8 | G6PD | Phenazopyridine |
| Cluster8 | G6PD | Methylene blue |
| Cluster8 | G6PD | Artesunate |
| Cluster8 | G6PD | Mefloquine |
| Cluster8 | G6PD | Dimercaprol |
| Cluster8 | G6PD | Glyburide |
| Cluster8 | G6PD | Rasburicase |
| Cluster8 | G6PD | Pegloticase |
| Cluster9 | GCLC | Sulfamethoxazole |
| Cluster4 | GNAS | Dobutamine |
| Cluster5 | GSTP1 | Isoniazid |
| Cluster5 | GSTP1 | Rifampicin |
| Cluster5 | GSTP1 | Dimethyl fumarate |
| Cluster5 | GSTP1 | Doxorubicin |
| Cluster5 | GSTP1 | Epirubicin |
| Cluster5 | GSTP1 | Thiotepa |
| Cluster5 | GSTP1 | Cyclophosphamide |
| Cluster5 | GSTP1 | Methotrexate |
| Cluster5 | GSTP1 | Oxaliplatin |
| Cluster5 | GSTP1 | Cisplatin |
| Cluster5 | GSTP1 | Fluorouracil |
| Cluster9 | HIF1A | Sorafenib |
| Cluster9 | HIF1A | Axitinib |
| Cluster9 | HLA-A | Carbamazepine |
| Cluster9 | HLA-A | Allopurinol |
| Cluster9 | HLA-A | Zonisamide |
| Cluster9 | HLA-A | Levetiracetam |
| Cluster9 | HLA-A | Lamotrigine |
| Cluster9 | HLA-A | Phenytoin |
| Cluster0 | HLA-B | Minocycline |
| Cluster0 | HLA-B | Methimazole |
| Continued | | |
| cluters | allmarkers.gene | drugName |
| Cluster0 | HLA-B | Dapsone |
| Cluster0 | HLA-B | Carbimazole |
| Cluster0 | HLA-B | Flucloxacillin |
| Cluster0 | HLA-B | Phenobarbital |
| Cluster0 | HLA-B | Ribavirin |
| Cluster0 | HLA-B | Sulfasalazine |
| Cluster0 | HLA-B | Oxcarbazepine |
| Cluster0 | HLA-B | Carbamazepine |
| Cluster0 | HLA-B | Acetazolamide |
| Cluster0 | HLA-B | Methazolamide |
| Cluster0 | HLA-B | Ticlopidine |
| Cluster0 | HLA-B | Clindamycin |
| Cluster0 | HLA-B | Abacavir |
| Cluster0 | HLA-B | Amoxicillin |
| Cluster0 | HLA-B | Allopurinol |
| Cluster0 | HLA-B | Lamotrigine |
| Cluster0 | HLA-B | Interferon beta-1a |
| Cluster0 | HLA-B | Interferon Alfa-2a, Recombinant |
| Cluster0 | HLA-B | Propylthiouracil |
| Cluster0 | HLA-B | Peginterferon alfa-2b |
| Cluster0 | HLA-B | Peginterferon alfa-2a |
| Cluster0 | HLA-B | Nevirapine |
| Cluster0 | HLA-B | Phenytoin |
| Cluster0 | HLA-B | Trichloroethylene |
| Cluster0 | HLA-C | Ribavirin |
| Cluster0 | HLA-C | Ustekinumab |
| Cluster0 | HLA-C | Carbamazepine |
| Cluster0 | HLA-C | Methazolamide |
| Cluster0 | HLA-C | Ticlopidine |
| Cluster0 | HLA-C | Allopurinol |
| Cluster0 | HLA-C | Peginterferon alfa-2b |
| Cluster0 | HLA-C | Nevirapine |
| Cluster0 | HLA-C | Phenytoin |
| Cluster0 | HLA-C | Methotrexate |
| Cluster5 | HLA-DPA1 | Peginterferon alfa-2b |
| Cluster5 | HLA-DPB1 | Acetylsalicylic acid |
| Cluster9 | HLA-DQA1 | Mercaptopurine |
| Cluster9 | HLA-DQA1 | Azathioprine |
| Cluster9 | HLA-DQA1 | Interferon beta-1b |
| Cluster9 | HLA-DQA1 | Interferon beta-1a |
| Cluster9 | HLA-DQA1 | Lapatinib |
|  |  |  |
| Continued | | |
| cluters | allmarkers.gene | drugName |
| Cluster9 | HLA-DQB1 | Flupirtine |
| Cluster9 | HLA-DQB1 | Amoxicillin |
| Cluster9 | HLA-DQB1 | Allopurinol |
| Cluster9 | HLA-DQB1 | Nevirapine |
| Cluster9 | HLA-DQB1 | Acetaminophen |
| Cluster5 | HLA-DRB1 | Simvastatin |
| Cluster5 | HLA-DRB1 | Pravastatin |
| Cluster5 | HLA-DRB1 | Atorvastatin |
| Cluster5 | HLA-DRB1 | Fluvastatin |
| Cluster5 | HLA-DRB1 | Rosuvastatin |
| Cluster5 | HLA-DRB1 | Flupirtine |
| Cluster5 | HLA-DRB1 | Oxcarbazepine |
| Cluster5 | HLA-DRB1 | Carbamazepine |
| Cluster5 | HLA-DRB1 | Mercaptopurine |
| Cluster5 | HLA-DRB1 | Allopurinol |
| Cluster5 | HLA-DRB1 | Azathioprine |
| Cluster5 | HLA-DRB1 | Interferon beta-1a |
| Cluster5 | HLA-DRB1 | Asparaginase Escherichia coli |
| Cluster5 | HLA-DRB1 | Nevirapine |
| Cluster5 | HLA-DRB1 | Lapatinib |
| Cluster5 | HLA-E | Adalimumab |
| Cluster5 | HLA-E | Certolizumab pegol |
| Cluster5 | HLA-E | Infliximab |
| Cluster5 | HLA-E | Etanercept |
| Cluster5 | HLA-E | Methotrexate |
| Cluster9 | HSP90AB1 | Bevacizumab |
| Cluster7 | HSPA1A | Carbamazepine |
| Cluster9 | HTRA1 | Ranibizumab |
| Cluster9 | HTRA1 | Bevacizumab |
| Cluster1 | IDO1 | Interferon Alfa-2a, Recombinant |
| Cluster1 | IDO1 | Peginterferon alfa-2b |
| Cluster0 | IGFBP3 | Fluorouracil |
| Cluster7 | IL6R | Tocilizumab |
| Cluster0 | ITGA2 | Acetylsalicylic acid |
| Cluster0 | ITGA2 | Clopidogrel |
| Cluster0 | KDR | Sorafenib |
| Cluster0 | KDR | Pazopanib |
| Cluster7 | MDM4 | Epirubicin |
| Cluster7 | MDM4 | Docetaxel |
| Cluster7 | NFIB | Methylphenidate |
| Cluster9 | NRP1 | Ranibizumab |
|  |  |  |
| Continued | | |
| cluters | allmarkers.gene | drugName |
| Cluster9 | NRP2 | Daunorubicin |
| Cluster0 | PERP | Epirubicin |
| Cluster0 | PERP | Cyclophosphamide |
| Cluster0 | PERP | Fluorouracil |
| Cluster5 | PLA2G4A | Atenolol |
| Cluster5 | PLA2G4A | Acetylsalicylic acid |
| Cluster5 | PLA2G4A | Diclofenac |
| Cluster5 | PLA2G4A | Acetaminophen |
| Cluster5 | PTGS2 | Ibuprofen |
| Cluster5 | PTGS2 | Atenolol |
| Cluster5 | PTGS2 | Acetylsalicylic acid |
| Cluster5 | PTGS2 | Rofecoxib |
| Cluster5 | PTGS2 | Oxaliplatin |
| Cluster5 | PTGS2 | Capecitabine |
| Cluster6 | RBX1 | Epirubicin |
| Cluster6 | RBX1 | Cyclophosphamide |
| Cluster6 | RBX1 | Fluorouracil |
| Cluster5 | RHOA | Simvastatin |
| Cluster5 | RHOA | Pravastatin |
| Cluster6 | RRM1 | Cladribine |
| Cluster6 | RRM1 | Cisplatin |
| Cluster6 | RRM1 | Gemcitabine |
| Cluster6 | RRM1 | Cytarabine |
| Cluster6 | RRM2 | Cladribine |
| Cluster6 | RRM2 | Cytarabine |
| Cluster7 | RXRA | Docetaxel |
| Cluster5 | SLC6A3 | Ethanol |
| Cluster5 | SLC6A3 | Clozapine |
| Cluster5 | SLC6A3 | Levodopa |
| Cluster9 | SOD2 | Heroin |
| Cluster9 | SOD2 | Cyclophosphamide |
| Cluster9 | SOD2 | Valproic Acid |
| Cluster9 | SOD2 | Asparaginase Escherichia coli |
| Cluster9 | SOD2 | Methotrexate |
| Cluster9 | SOD2 | Paclitaxel |
| Cluster7 | SPG7 | Thalidomide |
| Cluster7 | SPG7 | Docetaxel |
| Cluster7 | SREBF1 | Fluvastatin |
| Cluster7 | STAT3 | Isoniazid |
| Cluster7 | STAT3 | Streptomycin |
| Cluster7 | STAT3 | Ethambutol |
| Cluster7 | STAT3 | Rifampicin |
| Continued | | |
| cluters | allmarkers.gene | drugName |
| Cluster7 | STAT3 | Pyrazinamide |
| Cluster5 | TAPBP | Acetylsalicylic acid |
| Cluster6 | TOP2A | Epirubicin |
| Cluster6 | TOP2A | Cyclophosphamide |
| Cluster6 | TOP2A | Fluorouracil |
| Cluster6 | TYMS | Pemetrexed |
| Cluster6 | TYMS | Irinotecan |
| Cluster6 | TYMS | Raltitrexed |
| Cluster6 | TYMS | Methotrexate |
| Cluster6 | TYMS | Fluorouracil |
| Cluster6 | TYMS | Tegafur |
| Cluster6 | TYMS | Capecitabine |
| Cluster7 | UGGT2 | Hydrochlorothiazide |
| Cluster0 | VEGFA | Sildenafil |
| Cluster0 | VEGFA | Ranibizumab |
| Cluster0 | VEGFA | Pegaptanib |
| Cluster0 | VEGFA | Enalapril |
| Cluster0 | VEGFA | Cyclophosphamide |
| Cluster0 | VEGFA | Irinotecan |
| Cluster0 | VEGFA | Oxaliplatin |
| Cluster0 | VEGFA | Carboplatin |
| Cluster0 | VEGFA | Cisplatin |
| Cluster0 | VEGFA | Fluorouracil |
| Cluster0 | VEGFA | Sorafenib |
| Cluster0 | VEGFA | Sunitinib |
| Cluster0 | VEGFA | Leucovorin |
| Cluster0 | VEGFA | Capecitabine |
| Cluster0 | VEGFA | Bevacizumab |
| Cluster0 | VEGFA | Docetaxel |
| Cluster9 | VKORC1 | Warfarin |
| Cluster9 | VKORC1 | Phenprocoumon |
| Cluster9 | VKORC1 | Acenocoumarol |
| Cluster9 | VKORC1 | Fluindione |
| Cluster7 | WNK1 | Hydrochlorothiazide |
